# Planning with a gender lens: A gender analysis of pandemic preparedness plans from eight countries in Africa

**DOI:** 10.1016/j.hpopen.2023.100113

**Published:** 2023-12-12

**Authors:** Beverley M. Essue, Lydia Kapiriri, Hodan Mohamud, Marcela Claudia Veléz, Suzanne Kiwanuka

**Affiliations:** a Institute of Health Policy Management and Evaluation, Dalla Lana School of Public Health, 155 College Street, West Toronto, ON M5T 3M6, Canada; b McMaster University, 1280 Main Street West, Kenneth Taylor Hall Room 226, Hamilton, Ontario Postal Code L8S 4M4, Canada; c Department of Health Policy Planning and Management, Makerere University College of Health Sciences, School of Public Health, Uganda

**Keywords:** Gender, Gender analysis, Pandemic preparedness planning, Equity, COVID-19

## Abstract

**Background:**

Health planning and priority setting with a gender lens can help to anticipate and mitigate vulnerabilities that women and girls may experience in health systems, which is especially relevant during health emergencies. This study examined how gender considerations were accounted for in COVID-19 pandemic response planning in a subset of countries in Africa.

**Methods:**

Multi-country document review of national pandemic response plans (published before July 2020 and as of March 2022) from Ethiopia, Ghana, Kenya, Nigeria, Rwanda, South Africa, Uganda, and Zambia, supplemented with secondary data on gender representation on planning committees. A gender analysis framework informed the study design and the Morgan et al. matrix guided data extraction and analysis.

**Results:**

All plans reflected implicit and explicit considerations of the impacts of the pandemic responses on women and girls. Through a gender lens, the implicit considerations focused on ensuring safety and protections (e.g., training, access to personal protective equipment) for community and facility-based health care workers and broad engagement of the community in risk communication. The explicit gender considerations, reflected in a minority of plans, focused on addressing gender-based violence and providing access to essential services (e.g., sexual and reproductive health care, psychosocial supports), products (e.g., menstrual hygiene products) and social protection measures. Women were underrepresented on the COVID-19 planning committees in all countries.

**Conclusions:**

The plans reflected varying national efforts to develop pandemic responses that anticipated and reflected unique vulnerabilities faced by women, though subsequent plans reflected further consideration of gender-relevant impacts compared to initial plans. Embedding a gender lens in emergency preparedness planning furthers equity and could support anticipation and timely mitigation of negative outcomes for women and girls who are often further marginalized during health emergencies.

## Highlights

• Gender considerations are not consistently integrated into health system planning.
• Planning and priority setting with a gender lens can help to anticipate and mitigate vulnerabilities in health systems.
• COVID-19 pandemic preparedness plans in AFRO highlight implicit and explicit considerations of gender.
• A gender-lens in emergency planning enables decision makers to prioritize gender equity as a concomitant goal.

## 1 Introduction

The COVID-19 pandemic negatively affected all health systems, including in most countries in Africa. It stretched the resources of fragile health systems and threatened the ability of individuals to receive quality health services. The unpredictable nature of the pandemic exacerbated the circumstances of several groups of people, especially girls and women [1], who are often at risk of further marginalization during times of health and economic crises due to existing vulnerabilities and inequalities that are anchored in the social determinants of health [2]. For example, there is robust evidence emerging that women had a heightened risk of negative health and non-health outcomes including: reduced access to essential sexual and reproductive healthcare services [3], [4], increased risk of gender-based violence [5], including child marriage [6], sustained departures from the workforce [7], increased caregiving responsibilities [8] and a higher likelihood of school drop-outs [9]. The disproportionate impacts of the pandemic on women and girls reinforced the importance of being intentional in accounting for gender in emergency response planning.

Intentional consideration of gender and its impacts, it is argued, allows decision makers to anticipate the potential differential impacts of response measures within a population and plan to mitigate unwanted and unplanned consequences [10]. Gender is the social construction of the roles and characteristics of women, men, girls and boys [11]. Further, gender is not binary (i.e. girl/woman and boy/man), but rather exists on a continuum [11]. Gender can be a key driver of inequality in health systems because of the power dynamics that often determine who has what (i.e. access to resources); who does what (i.e. the division of labor and everyday practices); how values are defined (i.e. social norms, ideologies, beliefs and perceptions), and who holds power to decide (i.e. rules and decision-making) [12]. While there have been urgent calls to embed a ‘gender lens’ in policy development [13], [14], few studies have explored how gender has been accounted for in priority setting and planning for health, including during health emergencies.

Setting priorities during periods of uncertainty, such has health emergencies, can be especially challenging due to scant and evolving evidence and the heterogeneity of societal preferences and expectations on the tradeoffs associated with priority setting decisions [15], [16]. In times of emergencies, there is the added challenge of time constraints and the evolving nature of the context. Explicit criteria and broad stakeholder engagement are recognized as key elements of the process to guide priority setting and influence fair and equitable outcomes [17]. But there is emerging evidence that planning processes during the COVID-19 pandemic were not representative or reflective of the perspectives of key beneficiaries, including women and other populations who have been historically marginalized by disadvantage and underserved by health systems [18], [19]. Evidence on priority setting for the COVID-19 pandemic in the WHO African region revealed that few national plans defined priority populations or vulnerable populations in the initial pandemic response plans and that women, and access to essential health services, were identified or explicitly prioritized in only a minority of plans [20]; a finding that is consistent in many other countries around the world [21], [22].

Gender-inclusive planning can help to ensure that equity is at the forefront of how health resources are distributed and accessed during emergencies. While most countries in the WHO African region have implemented gender strategies to provide a bedrock on which to advance gender equality goals (Box 1), translating these values into health system planning, especially during times of crisis, is critical to ensure that nobody is left behind.

The objective of this study was to explore and describe, in a sample of countries in the WHO African Region, how gender considerations were accounted for in COVID-19 pandemic response planning. By investigating ‘gender-inclusive planning’, meaning the intentional or explicit consideration of gender equity in the formulation of pandemic responses, this study will contribute insights to support countries to strengthen emergency preparedness planning efforts as well as planning for more resilient health systems for the whole population.

## 2 Methods

### 2.1 Study approach

This is a secondary analysis of a large multi-country evaluation of priority setting for the COVID-19 response in 86 countries. The main priority setting study used the Kapiriri and Martin evaluation framework [24] to explore how decision-makers responded to the global pandemic and analyzed what decisions were made about the use of scarce resources including personal protective equipment, intensive care beds, vaccines, and other elements of the pandemic response and the prioritized populations. From the primary analysis of pandemic response plans from 18 countries in the WHO African Regional Office (AFRO) grouping [20], the authors found most of the reviewed pandemic plans included some aspects of priority setting. However, explicit equity considerations were detailed in only a few of the plans. For this secondary analysis, we focused on a sub-sample of eight countries (Ethiopia, Ghana, Kenya, Nigeria, Rwanda, South Africa, Uganda, and Zambia) that had plans written in English (Appendix Table 1).

We conducted a document review of the initial and subsequent pandemic response plans and a gender analysis of the content of the plans to explore how gender and its impacts were accounted for in the COVID-19 planning and response measures. We also examined the gender composition of the COVID-19 planning and response bodies in each country, using public data reported by the UN Women in the COVID-19 Global Gender Response Tracker [25], to explore the relationships between gender representation in leadership and gender-inclusive planning. In this gender analysis, we take a binary view of gender, noting its limitations but guided by the ways in which gender is captured in the pandemic plans in this region. We focus on the ways in which women and girls were accounted for, relative to men, in the priority setting for the COVID-19 response [26]. We note that this binary interpretation of gender does not capture the experiences and impacts of the pandemic response and priorities on gender diverse individuals which should be the focus of additional research.

### 2.2 Framework

The study design was guided by the Morgan et al. framework which explores how power is manifested to perpetuate and reinforce inequities; access to resources, roles, and practices; norms and beliefs; decision-making and autonomy, and policies, institutions, and laws [12]. We then used an adapted version of the Morgan et al. gender analysis matrix [10], and the Jhpiego et al. 2016 toolkit on gender analysis in health systems [27] to guide our data extraction and analysis. The main domains of the matrix enable an examination of how gender is accounted for in decisions about vulnerability to disease, exposure, response to illness/treatment, health system features (i.e. facilities and infrastructure) and economic, social and security impacts. For the purpose of this analysis, given our focus on priority setting for health emergencies, we adapted the matrix to focus on examining seven topic areas, namely: 1) policies, laws and institutions, 2) risk of exposure, 3) response to illness/treatment, 4) health system facilities and infrastructure, 5) economic impacts, 6) social impacts and, 7) security impacts, along four gender-relevant domains: i) access to resources; ii) distribution of labour practices and roles, iii) norms, values and beliefs and, iv) decision making power and autonomy).

### 2.3 Data sources

From the original sample of pandemic plans from the WHO African region [20], we included the initial pandemic response plans that were written in English and published between January 2020 and July 2020 (Appendix Table 1). We also retrieved the most recent plan, available as of March 2022 for each of these countries. Further, we supplemented data from the plans with information on gender representation in planning bodies from the UNWomen/UN Development Program COVID-19 Gender Response Tracker for each included country.

### 2.4 Data extraction

The plans were read by two independent reviewers in their entirety and the text was examined for how the response decisions explicitly and implicitly reflected considerations of pandemic-related risk and consequences for women and girls. Each plan was examined for the explicit mention of keywords such as equity, vulnerable people, girls, women, and gender. We also extracted information from the plans on the activities, resources and services that were outlined and discussed. Sections of text that aligned to each domain of the matrix were extracted verbatim as illustrative content and further summarized for the reporting. The matrix was populated for each plan (i.e. separate data extraction for the initial and most recent plans for each country).

### 2.5 Data analysis

The extracted data were assessed along each domain of the matrix using content analysis methodology [28] to explore and critique how a binary view of gender was reflected in the decisions and resources that were outlined and reflected in the plans. The first level of analysis entailed a critique of the initial plans for each country to examine how gender was accounted for in decisions about vulnerability to disease and exposure, response to illness/treatment, health system features (i.e. facilities and infrastructure) and economic, social and security impacts. We examined explicit and implicit considerations of gender in the decisions about the response and prioritized resources and critiqued the intentional and unintentional consequences. By this we mean that some decisions taken could be done so to intentionally mitigate negative outcomes or promote positive outcomes for women and girls. However, ‘gender-blind’ decisions do not intentionally consider the gendered impacts of decisions. In this scenario, it is possible that while gender-blind decisions could promote positive outcomes that benefit women (e.g., generally prioritizing supports for health care workers where the workforce are predominantly women), they can also worsen the situation for women/girls by not reflecting their diverse lived realities and circumstances (e.g., a prioritization of PPE for all health workers without accommodating for different sizes or access to PPE by all cadres of health workers).

We then compared the initial and most recent plans to assess whether the plans were updated to reflect attention to gender, given the evolving understanding of the gendered impact of the pandemic [4]. We conducted a cross-country analysis along the policy-relevant areas of the matrix to understand and distil learning on gender-inclusive planning during health emergencies among this sub-set of countries. The analysis was conducted by two researchers independently and then discussed and compared with the research team to align on the interpretation of the findings. Excel was used to manage the data extraction and analysis.

This study reviewed pandemic response plans that were available in the public domain and did not require ethics approval. However, the main study received ethics approval from McMaster University's Human Research Ethics Committee #MREB# 6468/.

## 3 Results

Of the eight countries evaluated in this study, all of the countries (except Zambia) released an updated plan during the study term, resulting in a total of 15 plans included in the analysis (eight initial plans and seven subsequent plans). In the sections that follow we present the results by each domain of the matrix and then discuss the implicit and explicit gender considerations that were reflected in the plans and describe distinctions between countries. A summary of the findings is outlined in Table 1 and a detailed overview of the findings for each country is provided in Appendix Table 2.

**Table 1:** Summary of main findings from gender analysis of pandemic response plans from eight countries in the WHO African Region.

Pandemic Response Domains	Gender Analysis Domains			
	Access to Training and Resources	Distribution of Labour, Practices, Roles	Norms, Values, Beliefs	Decision-making Power and Autonomy
Policies, Laws, Institutions	–		–	National COVID-19 task force housed within the Ministry of Health with aligned budget *[All plans]*. Called for the protection of vulnerable populations, including women *[Kenya, Nigeria]*. *Explicit* Under-representation of women on COVID-19 response committees *[All plans; Implicit]*
Risk of Exposure	Ensure hand washing and disinfection facilities and supplies in the community, schools, workplaces and hospital / health facilities. *[All plans; Implicit]* Ensure training for HCW (rapid-response teams, infection, exposure, and control, etc), access to necessary protective equipment (e.g., PPE) and support services (e.g., mental health, psychosocial supports and counseling resources) to reduce the risk of exposure and transmission of COVID-19. Provide safe work environments. Establish training and resources on risk of exposure but plans often lacked specificity on how to tailor the training and target populations. *[All plans; Implicit]* Strengthen the capacity and ensure remuneration of homecare workers *[Ethiopia, Ghana Rwanda; Implicit]*	Enhance training and support for all cadres of health workers, including rapid response teams, laboratory staff, homecare workers, community health workers to ensure that HCWs are trained and supported to contribute and engage effectively and safely in the risk control efforts *[Kenya, Rwanda Uganda, Zambia; Implicit]* Expand roles for reporting exposures in the community (e.g., head of household report on behalf of the household) *[Uganda. Implicit]*	Engage community leaders to support risk communication and influence community norms, values, and behaviours about COVID-19 *[Ethiopia, Ghana, Kenya, Nigeria, Rwanda; Implicit]* Develop strategy for community engagement and distribution of information on preventative measures *[Nigeria, Zambia, Uganda, Kenya. Implicit]*. The importance of tailoring messaging to vulnerable groups is identified in few plans [*Kenya*: Preparing messages through a participatory process to ensure that messaging is effectively targeted to the public including at-risk groups; *Explicit*] Detect and respond to misinformation on COVID-19 within communities *[Kenya, Implicit]* Gather information on community beliefs and feedback to inform gaps in the current plan *[Kenya; Implicit]* Hold public dialogue discussions and engagements aimed to reach those who are not accessing information through other channels. Women and girls are not called out specifically in the groups to be engaged *[Kenya; Implicit]*	Develop communication strategies, guidelines and content for different audiences, to ensure that communities are being reached and reduce the risk and spread of COVID-19 among the most vulnerable who may not access information using usual fora *[Ethiopia, Ghana; Explicit]* Implement national risk-communication and community engagement plan for COVID-19, including details of anticipated public health measures *[Zambia; Implicit]* Support community control through school closures and other measures to control the spread of the virus and associated impacts for over-burdened health systems and HCWs. This negatively impacted women, who faced increased care and home-schooling responsibilities, and girls, who had higher school drop-out rates. *[Ghana; Implicit]* Implement community-based hygiene and social distancing policies, relevant for those working in informal and precarious work *[Ghana; Implicit]*
Response to Illness/Treatment	Educate staff and provide critical resources to respond to surges in COVID-19 infections *[Ethiopia, Uganda; Implicit]* Ensure testing, identification, and quarantine measures for HCWs to control the spread of COVID-19 and ensuring access to these measures by vulnerable groups in the community *[Ethiopia, Ghana, Zambia; Explicit]* Prioritise data collection to support ongoing response efforts and learning. The plans did not indicate disaggregating data by sex/gender. *[Rwanda, Uganda; Implicit]* Ensure adequate financial support and trained human resources for vaccine supply, delivery, monitoring, and evaluation. *[Rwanda, Uganda; Implicit]* Map populations of those most impacted by the pandemic to ensure efforts / resources are in place to address the needs of these groups. *[Zambia; Implicit]*	Provide technical and operational support through short to medium term secondment and deployment of staff to address the workload challenges for frontline workers. Support (through training) HCWs working in new roles. This also potentially contributes pressures elsewhere in the system. Also potential for unintended consequences from change in role / increase in workload for redeployed staff *[Zambia, Uganda; Implicit]* Plan for health workforce impacts of increased demands on the system and address surge capacity. Anticipate and address workforce shortages *[Ethiopia; Implicit]*	–	Monitor the quality of COVID-19 surveillance data and interventions which is used to support planning and targeted responses *[Rwanda; Implicit]* Establish approaches to support care of the most vulnerable groups and key populations, including defining who these populations are (e.g., prisoners, refugees, the elderly, those who comorbidities, children in care institutions, women etc.) *[Rwanda; Explicit]* Conduct a rapid assessment of the communities who are most impacted in terms of organization; health behaviour and social protection measures; and occupational health and safety to adjust the response to account for the potential differences in needs within the population [*Uganda Explicit]*
Health Systems: Facilities and Infrastructure	Identify resources requirements for continuity of essential services [*All plans; Implicit]* Identify and ensure access to essential products (e.g., female hygiene products) *[Zambia]* and equipment (e.g., PPE) *[All plans, Explicit].* Conduct ongoing training on COVID-19 case identification, triage, reporting, contact tracing, infection prevention and control, etc. for all health facility staff; train frontline healthcare workers on sample collection for screening and surveillance activities. *[Rwanda, Uganda and in all subsequent plans; Implicit]* Provide psychosocial supports to health care workers to mitigate and address burden and burnout *[Uganda, Zambia; Implicit]* Ensure access to health care by reducing out of pocket payments for patients or expanding health insurance coverage and benefits *[Zambia; Implicit]*	Engage and support community health volunteers and commodity distributors by ensuring that they are oriented to the prevention of COVID 19 and ensure that they have adequate PPEs and supplies [*Zambia; Implicit]*	–	Identify and ensure continuity of essential services *[All plans; Implicit]* Create databases and ensure disaggregated data is available to understand critical elements such as hotspots and most vulnerable population groups *[Zambia; Explicit]* Document lessons learned to inform future preparedness and response activities as well as the production of storytelling archive of pictures, stories, and narratives of all steps of the outbreak *[Zambia; Implicit]*
Economic Impacts	Provide social protection and income protection measures, including in communities of need, to mitigate the health and non-health impacts of the pandemic and target to those in greatest need. *[Uganda, Zambia; Implicit]* Engage with local donors and existing programmes to mobilize/allocate resources and capacities to implement operational plan to support vulnerable populations, including women and girls (among other groups), to deal with the negative impacts of the pandemic *[Zambia; Explicit]* Ensure access to food and non-food items *[Zambia; Explicit]* Providing health packages to those who experience gender-based violence to mitigate the impacts. Focus here is on supporting victims, not prevention of the violence *[Uganda; Explicit].*			All plans outlined lock-down and shelter-in-place policies. But no plans considered the potential for disproportionately negative economic outcomes by the sectors and industries that were hardest hit and how this impact might differ between genders. [*Implicit.]* Strengthen community engagement and social protection structures for COVID-19 in communities to mitigate the impact of the ‘shocks’ caused by COVID to the economy *[All plans; Implicit]*. Provide capital/financial support to those who experience gender-based violence to mitigate the impacts. Focus here is on supporting victims, not prevention of the violence *[Uganda; Explicit].* Identify critical measures to ensure safety of working environments to limit economic impacts of closures and lockdowns *[Zambia; Implicit]*
Social Impacts	Conduct a rapid assessment of COVID-19 impacts on gender-based violence against children, Emergency Alternative care and provisions for children in detention, to inform development of an evidence based prevention and Response Plan and policy briefs on GBV/VAC *[Uganda; Explicit]* Ensure social supports, such as nutritional support and emergency support services for orphans and vulnerable children *[Uganda; Implicit]* Implement measures to enable transitions to online school (e.g. materials for distance education; radio schooling) *[Ethiopia, Kenya, Rwanda, South Africa, Uganda; Explicit]*	Provide services and alternate supports for those impacted by gender-based violence, violence against children, children without parental care and juvenile offenders, to reduce negative social impacts of COVID-19 on women and children *[Zambia; Explicit]*	Develop community engagement for prevention of violence, discrimination, marginalization and xenophobia through promotion of social cohesion messaging and activities *[Zambia; Explicit]*	Access to basic subsistence during the lockdown periods *[Ethiopia, Ghana, Nigeria, Kenya, Rwanda, Zambia]. Implicit* Enforce self-quarantine guidance. But failures to provide social supports (e.g., financial support for missed wages/work, childcare, etc.) had the potential to negatively impact women and girls *[Rwanda; Implicit]* Establish a national risk communication and community engagement plan that accounts for health and non-health consequences *[Rwanda; Implicit]* Document lessons learned to inform future preparedness and response activities. Note: Enables learning that can be updated in future plans *[Rwanda; Implicit]* Collect disaggregated data on impacts/ consequences/ outcomes to learn about the impact of the pandemic and response on vulnerable groups in the population *[Zambia; Implicit]* Develop a Response Plan and strategy for gender-based violence and violence against children related to COVID-19 *[Uganda; Explicit]*
Security Impacts	–	–	–	The security impacts detailed in the plans did not reflect implicit or explicit considerations of gender. Plan to collect sex-based data as part of quarantine reporting and for foreign travelers during points of entry *[Nigeria; Explicit]*

(-) denotes no explicit or implicit consideration of impacts that might affect women and girls in the COVID-19 response plans reviewed.

The plans in which this domain is addressed are noted in square brackets: [country].

Implicit: This indicates that while the plans did not explicitly specify that the response was intended to mitigate impacts for women and girls specially, there is a perceived implicit impact (positive or negative). For example, given that women are the majority of health care workers (HCWs), responses that target HCWs stand to benefit women by mitigating the impacts for women in these roles.

Explicit: Here women and/or girls are called out specifically as a target for the response.

### 3.1 Policies, laws and institutions

Seven out of eight initial plans described government roles and responsibilities for spearheading prevention and control of the COVID-19 pandemic. For Nigeria and Kenya, their COVID-19 plans described the need for a National COVID-19 task force housed within the Ministry of Health with executive power to mobilize various stakeholders including technical partners, other government agencies, and development partners to reduce the vulnerability of certain groups, including women. Similarly, Ghana developed a COVID-19 response plan through a whole-of-government and whole-of-society approach. This plan was developed in alignment with the a country’s long-term strategy for achieving universal health coverage, which seeks to ensure that all groups of people – including those who are marginalized by disadvantage receive quality healthcare services. The plan for Rwanda described the importance of conducting an initial capacity assessment and risk analysis, including mapping of vulnerable populations for targeted support.

On review of the subsequent plans, policies, laws, and institutions were critical for ensuring the continuation of access to services during the lockdown periods of the COVID-19 containment strategy in each country, including access to essential health services for women with preexisting conditions and for sexual and reproductive health needs. In the majority of countries (n = 6), there were initial and sustained policies that ensured access to basic subsistence during the lockdown periods. While not explicitly calling out the benefits for women, policies that address subsistence needs were important for girls and women who were disproportionately impacted by lockdowns that restricted participation in informal work. Informal work is defined as all remunerative work that is not registered, regulated or protected by existing legal or regulatory frameworks, and typically lacks secure employment contracts, workers' benefits, social protection or workers' representation [29]. This has relevance in this sub-set of countries where women and girls account for between 37% (South Africa) and 88% (Ghana) of the work in the non-agricultural informal sectors in these settings [25].

On review of the membership of the initial COVID-19 planning bodies, none had gender parity in the composition of the membership, highlighting an absence of key perspectives in the development of the responses (Table 2) [25].

**Table 2:** Gender composition of the committees.

Country	Percentage of Committee That Are Women
Ethiopia	38 %
Ghana	N/A
Kenya	19 %
Nigeria	17 %
Rwanda	N/A
South Africa	38 %
Uganda	20 %
Zambia	N/A

Note: N/A means this information was not reported by the UNDP gender tracker.

### 3.2 Risk of exposure

All of the plans described risk communication measures and resource mobilization efforts aimed at reducing exposure risk for the full population. These strategies reflected both implicit and explicit consideration of gender and stood to impact women and girls. For example, the plan from Zambia called for vulnerable groups, including women, to avoid exposure to the virus and the Ministry of Health played a critical role in disseminating information on the risk of exposure in areas that were more likely to be occupied and frequented by women, including in healthcare centers, childcare centers, and residential care homes for the elderly. The plan from Ethiopia specified the need to collect data on the rate at which pregnant women were exposed to the virus to ensure that antenatal evaluations for pregnant women with COVID-19 could be safely postponed.

Most plans mentioned community engagement strategies to devise risk communication approaches. In the case of Zambia, the COVID-19 response plan sought to leverage the power and influence of chiefs and community leaders to mobilize people and encourage behavior change as a means of preventing the spread of the virus but also as an approach to reducing vulnerability for certain groups and populations. Further, the plan for Ethiopia indicated healthcare workers were encouraged to connect with women about their risk of exposure and support them by making connections with religious services and leaders in the community.

All eight of the initial pandemic response plans described the importance of providing both infection prevention and control training to healthcare workers, as well as appropriate personal protective equipment (PPE) to healthcare workers. Community health workers were prioritized in several plans given their involvement in the frontline response and management of the pandemic and thus, faced a higher risk of exposure. For example, the initial COVID-19 plan from Nigeria described issuing PPE to personnel in all sectors, including community health workers. While not explicit, by prioritizing protections for frontline health care workers, including community health workers, who are predominately women [31], women received some of the necessary protections to mitigate the risk of contracting and spreading COVID-19, enabling safer working conditions. The plan for South Africa described making it a priority to provide appropriate PPE for all healthcare workers with monitoring in place to ensure there was equal distribution across facilities.

Further, the plans from Uganda and Zambia described provisions to ensure PPE (i.e. masks, gloves, etc.) were available to individuals working and caregiving outside of healthcare roles, in the wider community. This reflects implicit consideration of the multitude of roles that women play in supporting communities and families, including support and caregiving for sick children and the elderly.

In the updated plans, there was little change in how women’s risk of exposure was reflected. A notable exception was the plan from Uganda. In the original plan, it was posited that communities often lacked the resources to ensure protection against the virus. However, the updated plan defined a role for the heads of households to report COVID-19 exposures directly to the government. This had the potential to reduce the agency of women to report on their own health status and at the same time, marginalized households with single females. Further, Nigeria’s updated plan identified a coordinated supply of PPE for healthcare workers and suggested continuous training on the proper use of PPE through workplace reminders, as well as tracking of PPE. Through the provision of adequate and appropriate PPE, there were plans to help reduce the risk of exposure to the virus by female healthcare workers.

The updated plans from Uganda and Rwanda identified a need for greater attention and support to mitigate the risk of exposure in the most vulnerable populations (i.e prisoners, refugees, the elderly, etc.). This reflects implicit considerations of gender as women and girls are disproportionately represented in refugee, elderly and low socioeconomic populations in these settings.

### 3.3 Response to illness/treatment

The plans reflected both implicit and explicit consideration of gender in the measures identified to respond effectively to COVID-19. The implicit considerations focused on efforts to recruit, educate, and support the health workforce needed to anticipate and respond effectively to COVID-19 infections (Ethiopia, Ghana, Uganda, Zambia). The plans from Zambia and Uganda supported the need for short to medium term secondment and deployment of staff to anticipate and address workload challenges among frontline workers. The plans from Rwanda and Uganda also discussed ensuring adequate financial support more generally for human resource requirements as well as vaccine, delivery, monitoring, and evaluation. Gender is seen as an implicit consideration here given the higher participation of women in the health sector and thus, involved in the COVID-19 response.

The explicit considerations of gender were more evident in the updated plans and focused on ensuring testing, identification, and quarantine measures for HCWs to control the spread of COVID-19 and ensure access to these measures by vulnerable groups in the community, including women and children (Ethiopia, Ghana, Zambia). The plans from Zambia and Uganda discussed the need to map the populations most impacted by the pandemic to ensure resources were in place to address the needs of these groups. While several plans outlined the need for data collection to support ongoing response efforts and learning (Rwanda, Uganda), few mentioned the need for these data to be sex or gender disaggregated.

The plan from Rwanda established approaches to support care of the most vulnerable groups and key populations, including identifying prioritiy populations (e.g., prisoners, refugees, the elderly, those who comorbidities, children in care institutions, women etc.).

### 3.4 Health systems: facilities and infrastructure

Beyond the risk of exposure and infection, healthcare workers experienced an increased mental health burden from increased demands associated with implementing and supporting the COVID-19 responses. There was evidence that women experienced a greater mental health toll [4]. The psychosocial impacts of the pandemic were highlighted in all but one (Rwanda) of the initial pandemic plans, and these plans outlined a need to ensure psychosocial counselling and support to healthcare workers. The plans from Uganda and Zambia also raised the importance of providing psychosocial supports to those impacted by COVID-19 outside of healthcare workers, including families of health care workers and those who experienced COVID-19-related deaths.

Ethiopia’s updated plan described the need to establish contingency plans in case of staff shortages, to ensure staffing levels could support surge capacity and a guide to manage increasing staff illness and work leave requests. These workforce policies supported safer working conditions for all healthcare workers, and thus had implicit benefits for women in these positions.

Beyond healthcare workers, all plans reflected the urgency in ensuring access to primary healthcare services. There was a focus on decentralizing health services in the plans from Zambia, Rwanda, Ghana, Kenya, Uganda, and South Africa. The plan from Uganda indicated decentralized services provided wider coverage and improved access, especially for women and girls, reflecting explicit consideration of gender. The plans for Ghana and Kenya also called for a decentralized approach to health service delivery to support better outcomes for target populations. The revised plans for South Africa and Ghana reflected calls for further decentralization to support the COVID-19 resurgence strategy.

The plans from Zambia and Rwanda prioritized ensuring access to sexual and reproductive health services, reflecting an explicit consideration of women and girls’ health needs during the pandemic. In the plans from Zambia, the focus was on creating community structures to deliver sexual and reproductive health commodities to maintain services during the pandemic. The plan from Rwanda explicitly suggested the need for women to have access to long-lasting insecticide nets, and for health services for HIV, tuberculous and malaria to be maintained. Whereas the plan from Kenya outlined the need to provide lifesaving services, but it was not explicitly mentioned whether women were prioritized.

In the plan from Uganda, the government prioritized the needs of women and children by ensuring that there was continuity of essential care – including for those suffering from chronic conditions and explicitly called out the need for the government to prioritize women’s access to child and maternal healthcare. In South Africa’s case, the ability for women to access key services depended on their level of intersectionality, particularly, being elderly (i.e. minimum age not defined) and living with comorbidities. Nigeria’s updated COVID-19 plan focused on the bureaucratic/administrative aspects of service delivery with little mention of how essential services for women should be continued, gender-based violence combated, and how girls/women could be mobilized.

The importance of establishing databases with disaggregated data by sex and gender was described in the updated plan from Zambia to support ongoing COVID-19 planning and response but this was not mentioned explicitly in the other plans.

### 3.5 Economic impacts

Government measures used to mitigate the spread of COVID-19, such as mandatory lockdowns and isolation policies, had significant economic and social consequences. Seven out of the eight countries (excluding South Africa) highlighted the economic impacts of the COVID-19 pandemic in their response plans, but none described the potential for negative economic consequences to be disproportionately experienced by workers in the sectors that would be most affected (e.g., service, tourism, informal workers); sectors which also reflect high participation by women. In three plans (Ethiopia, Uganda and Zambia), economic supports (e.g., the provision of packages containing food and basic items), were targeted to support vulnerable populations and women and girls were either implied or specified. Furthermore, the updated plans from Ethiopia, Ghana, Nigeria, South Africa and Uganda outlined economic relief programs (e.g., social cash transfers) and income protection measures in response to the income and job losses associated with prolonged lock downs in each country. Zambia had a comprehensive plan to mitigate the economic burden of the pandemic, which included increasing the cash transfer amounts to people receiving social assistance prior to the pandemic, the use of mobile payment systems to prevent disruptions in the delivery of cash transfers, and the provision of food to communities. A revision to the initial plan from Ghana saw the establishment of a COVID-19 Trust Fund ($40 million dollar fund) to provide people with food packages and hot meals during the lockdown periods. However, there are sparse details in the plan on the eligibility criteria for accessing the fund. Moreover, Zambia leveraged the United Nations Children’s Fund to supplement its social cash transfer program to reach female-headed households with children. These measures stand to benefit women if appropriately targeted but there were few details on how eligibility for the programs was established in each setting.

Protective policies for healthcare workers, including sick leave were rarely mentioned within the initial pandemic response plans. Ethiopia was the only country that discussed sick leave and hazard pay, highlighting the importance of providing healthcare workers sick leave and compensation for the increased burden of their work. As above, the measures targeted to health care workers reflected implicit gender-relevant considerations.

### 3.6 Social impacts

The shelter in place policies required to contain the COVID-19 pandemic placed women and children at a heightened risk of experiencing violence in the home and globally, illuminated the shadow pandemic of gender-based violence that pre-dated the COVID-19 pandemic [34]. Accordingly, most of the social impacts reflected in the plans outlined an explicit focus on mitigating the risks of gender-based violence and providing support for victims. The initial plans from Uganda, Kenya, Ghana, and Zambia, described concerns about the increased risks of exposure to gender-based violence to varying degrees and in some cases separate guidelines were developed to outline focused action to mitigate the risks of gender-based violence (e.g., Uganda, Zambia). For example, the plan from Zambia explicitly defined preventive measures and supports available to those impacted by gender-based violence, including orienting community volunteers to provide anti-gender-based violence messaging within communities and the continuation of social welfare services to support those impacted. Similarly, in the plan from Kenya, the Ministry of Health defined gender-based violence as being a heinous crime and described measures to reduce the vulnerability of women and girls. The plan from Ghana focused on the need to counteract gender-based violence to ensure that the COVID-19 pandemic was not worsening the quality of life for vulnerable people such as girls and women. An amendment to the initial Zambian plan focused on leveraging the Safe Motherhood Action Groups (SMAG) platform as a means of reporting any sexual exploitation issues that women and girls were facing and to better monitor and address concerns about increases in incidents of GBV in the country.

While Uganda’s plan recognized that the pandemic could exacerbate the likelihood of gender-based violence, the plan also called for suspensions to court services, in line with the general policies to halt public gatherings, meetings, and prayers in churches and mosques; eliminating essential legal protections for victims. Additionally, the heads of households were mandated to report COVID-19 cases to the government which established another potential means for perpetrators to exert control over their partners in the home.

During mandatory lockdowns schools were required to close in all countries studied. This had significant impacts on both women and girls, as evidence suggests the closure of schools put girls at an increased risk of sexual violence, child marriage, adolescent pregnancy and permanent departures from education, which disproportionately affected disadvantaged and vulnerable girls [32]. Five countries discussed school closures within their plans (exceptions were South Africa, Ethiopia and Kenya). Only three of the five plans outlined supports for school children: Zambia discussed the provision of distance education to all learners (especially the disadvantaged), Rwanda discussed the development of radio education programming and home-schooling efforts to maintain continuity of education, and Uganda discussed the use of radio education programming.

Additionally, school closures resulted in an increased burden of unpaid work for both women and girls, in part due to widespread norms and practices that view unpaid work as the responsibility of women and girls [33]. None of the plans discussed specific supports to address the impact of school closures on women.

### 3.7 Security impacts

Most of the plans did not examine the security impacts of the COVID-19 pandemic with a gender lens. Nevertheless, there was a focus on limiting travel and the extent to which individuals moved in and out of their respective countries. A minority of plans focused on the need to strengthen the health infrastructure to recognize and support community movement and migration, including ensuring appropriate levels of disease surveillance at borders. Notably, Kenya’s COVID-19 plan called out the potential for heightened risks for refugee communities or those situated at borders. However, there was little discussion of the potential impacts of these travel restrictions on sub-populations, including women.

Nigeria’s subsequent plan indicated the need for sex-based data collection to be integrated into quarantine reporting to better monitor the impact of the pandemic. In addition, foreign travelers who entered Nigeria had data on their sex collected during points of entry.

### 3.8 Lessons on gender-inclusive planning for emergencies

There was variability in how gender was reflected across the initial pandemic response plans. In the initial plans, gender considerations were reflected mostly in two of the four gender analyses domains (i.e. a) access to resources; b) decision-making, power, and autonomy). Within these two areas, there was commonality across the plans with a focus on addressing the prevention of the virus and reducing the risk of transmission through the distribution of PPE, providing psychosocial support to both healthcare workers and patients, providing cash transfers to vulnerable populations (i.e. elderly), combating gender-based violence and the continuation of essential health services (i.e sexual health services). In the other two policy areas (i.e., c) the distribution of practices and roles and, d) the norms, values and beliefs, we did not find evidence of strong considerations of gender in the initial pandemic response plans.

In the subsequent pandemic response plans, there was evidence of enhanced gender considerations. However, the improvements are not equal across all of the topic domains. For example, there is evidence of gender considerations in the following domains: system facilities and infrastructure, economic impacts, and social impacts. Examples included providing healthcare workers with sick leave, as well as recognizing the impact of COVID-19 on family caregivers by providing an increase in material supports (i.e. PPE and psychosocial support).

When looking across the countries and between the initial and subsequent plans, most of the evidence on gender-inclusive planning is implicit rather than explicit signaling opportunities for more systematic contemplation of gender within pandemic planning.

Based on the findings from this study, we propose a list of considerations for emergency planning with a gender lens to support ongoing health emergency preparedness and response planning (Table 3).

**Table 3:** Considerations for emergency response planning with a gender-lens.

Pandemic response domains	Relevant considerations
Policies, Laws, Institutions	- How are gender and other diverse stakeholder perspectives meaningfully included on planning and response committees? ○ What key perspectives are missing from the decision-making process? - What existing advisory bodies and processes can be engaged to include gender-relevant community perspectives from in the decision-making processes? - What are the intended and potential unintended consequences of emergency response policies and guidelines for the most marginalized or vulnerable populations, including women and girls? ○ How are these monitored? ○ What resources are availed to mitigate negative consequences and ensure all individuals benefit from the response strategy? - How does the planning process effectively engage other sectors (e.g., health, education, labour, social protection, private sector (tourism, sales), etc) where there are gender-relevant differences in participation to develop a coordinated response?
Exposure	- Which roles and functions (formal or informal); implicit or explicit) face: ○ increased vulnerability or risk of infection; ○ barriers to accessing health and social services? ○ increased risk of negative outcomes? *How do gender and other intersectional dimensions exacerbate the risk of exposure, response to illness/treatment and outcomes?* - What populations and resources are prioritized (explicitly or implicitly) in the emergency response? - Which disaggregated data can be used to monitor risk, response to illness, access barriers and outcomes within populations? ○ How can these data be used to inform ongoing response and preparedness planning? ○ What impact(s) do the response(s) have on existing gender inequalities?
Response to Illness/Treatment	
Health Systems: Facilities and Infrastructure	- Which populations / communities are typically under-served in health systems in the given context? - What different strategies can be used to ensure infection, prevention and control, risk communication, testing and treatment strategies reach communities and individuals who are often under-served by health systems, including women and girls? - How is gender accounted for in the criteria used to prioritize essential services, resources and populations? - Which institutions/stakeholders/workforce should carry the responsibility for implementing the response strategies and how is gender accounted for in the approaches for training, protection, support, and compensation?
Economic, Social and Security Impacts	- How is gender accounted for in the criteria used to determine essential work? - What are the potential economic, social, legal and safety consequences that could result from the emergency response strategies and do they stand to impact existing gender inequalities? - What resources are availed to mitigate the impacts on vulnerable populations?

Questions were developed based on the findings from this research and build from previous literature: [10], [12], [27].

## 4 Discussion

This study examined the initial and subsequent national COVID-19 plans from a subset of eight countries from the WHO African region to explore and critique how gender was considered in national-level planning for the COVID-19 pandemic response in each country. The pandemic response plans reflected varying levels of implicit and explicit consideration of gender and gender impacts, as well as evidence of increased attention to gender in the subsequent plans. This study highlights an opportunity for a more comprehensive and systematic integration of a gender lens in planning to support pandemic and health emergency preparedness in the eight countries and demonstrates the application of a gender-analysis matrix that can be used to support and systematize this process. Additionally, it contributes to a growing body of literature on the importance of pandemic responses as a key opportunity to promote gender equity and redress the inequities and vulnerabilities experienced by women and girls that are often exacerbated during times of crises [2].

Women and girls experienced disproportionate health, economic, and social impacts from the COVID-19 pandemic in part due to pre-existing structural inequities and gender norms [4]. Women were overrepresented in sectors and roles that were most strongly impacted in the pandemic, including healthcare [38], as well as low-wage and the informal sector jobs [30]. Within the health system women were placed at a higher risk of exposure to and infection with COVID-19. To mitigate this risk all eight of the pandemic response plans highlighted the importance of providing both infection prevention and control training, as well as appropriate PPE to healthcare workers and to women outside of healthcare roles; a decision that stood to have important impacts on mitigating the risk of exposure for a majority of the female health workforce in the subset of countries studied. Beyond risk of exposure and infection, healthcare workers also experienced an increase in their mental health burden [4]. Yet only a minority of plans explicitly addressed the provision of psychosocial supports for healthcare workers.

While most plans highlighted the economic impacts of the COVID-19 pandemic, only a minority of plans addressed sick leave or other income support provisions. Economic relief in the form of a cash transfers and income supports were an important means to relieve the socioeconomic burden of the pandemic [36] but these measures were not universally available across sectors in all study countries [34]. Income supports and cash transfers were especially relevant for the many women working in low-wage informal sectors in each of the study countries. These women faced increased vulnerability to poverty and disempowerment in the absence of arrangements to maintain wages during periods of lockdowns and illness. Evidence from the initial stages of the pandemic showed a decrease in the income of more than 700 million women working in informal economies around the world during the first month of the pandemic and 72% of domestic workers (80% of whom are women) losing their jobs as a result of COVID-19 [35]. Economic relief in the form of cash transfers and income supports was a key means to relieve the socioeconomic burden of the pandemic [36] especially when paired with other policies such as paid sick leave arrangements that was especially relevant for healthcare workers in all contexts.

Much has been written about the importance of representation in leadership, including during health emergencies as key for promoting and implementing gender-inclusive planning [39], [40], [41]. It is posited that such equal representation supports planning that takes account of intentional and unintentional impacts of decisions on the whole population, including during health emergencies [42]. We found an underrepresentation of women on planning bodies for the countries with available data. While these findings reflect the committee composition during the COVID-19 pandemic, it is likely, as previously reported [18], that prior experiences and commitments to gender representation in leadership and planning influenced the committee compositions during the pandemic in each setting [37]. This emphasizes the need for investment in efforts to foster equitable representation of women within health system governance to support gender-inclusive planning during times of emergencies and crises.

A strength of this study is that it provides an in-depth analysis of the ways in which gender was considered in the COVID-19 plans and responses in several countries in the WHO African region. The analysis highlights strengths across the national plans as well as some blind spots in emergency planning that could negatively impact outcomes for women and girls. We used a published gender analysis framework and matrix to examine health emergency priority setting and planning over time and to explore how gender factors into decision making and resource allocation during emergencies. This study also integrates gender analysis with the evaluation of planning efforts and distills opportunities to explicitly account for gender inequity in ongoing health system planning efforts.

However, this study also has limitations. We focus on information from publicly available plans and do not account for insights from civil society and the public in this analysis. Thus, we assessed what was planned and in the next phase of the research, we will use key informant interviews, including with members of the public, to explore perspectives and experiences of implementing the plans and their impact on women’s health, economic and social outcomes. We examined national plans however, in some countries, these plans would be supplemented with sub-national planning and implementation strategies that could contain specifics on gender relevant priorities. Finally, and importantly, we adopt a binary view of gender in this analysis and focus on providing an in-depth analysis of how women’s health, needs, and experiences are reflected in the plans. A broader analysis that accounts for the spectrum of gender diversity would yield key insights for subgroups that have also been marginalized in the pandemic responses.

## 5 Conclusion

During the COVID-19 pandemic, countries were confronted with understanding how to provide key services and supports to their populations in an equitable and timely fashion. This study highlighted efforts to develop pandemic response plans that anticipated and reflected unique vulnerabilities faced by women and girls in the subset of countries investigated. The application of gender-analysis to pandemic preparedness planning suggests that a gender lens was not systematically applied across all plans. However, as the plans developed over the course of the pandemic, there was further consideration of gender-relevant impacts evident in the plans. Pandemic response planning with a gender lens, including through collaborative processes at the highest levels of planning, stands to explicitly embed equity in emergency preparedness planning. This can support more timely anticipation and mitigation of negative outcomes for women and girls while furthering gender equity in health systems as concomitant goals

Text box 1: Summary of a selection of commitments to gender equality in a subset of national gender plans from the WHO African region There has been a demonstrated commitment to advance gender equality in the WHO African region, with national gender plans or strategies now in place in most countries that link global and national priorities and provide a roadmap to achieve gender equality goals. In most national gender strategies, gender equality goals are defined as a collaborative effort of the government and cross-sectoral stakeholders including civil society and the private sector. The national gender plans and strategies articulate a role for governments to either form strategic partnerships (i.e. Nigeria, Ghana, Zambia) or use constitutional obligations to prioritize gender (i.e. Uganda, Rwanda, South Africa, Kenya, Ethiopia).

Despite stated and legislated commitments for prioritizing gender, implementation gaps have hindered progress in many settings. For example, most national gender strategies have commitments and targets for combating gender-based violence (GBV) (i.e. Ghana, Kenya, Rwanda, Nigeria, Uganda, and Zambia), yet the prevalence of GBV remains high [22], with inadequate resource allocation to support GBV service implementation in most settings [23].

Representation in the legislature can also be a key element of a country’s gender strategy. For example, in Rwanda, the constitution mandates that 30% of parliamentary seats should be occupied by women and this has been attributed to improved health standards for women, as well as improvements in access to affordable, quality health services [24]. While in Ethiopia, women’s representation in parliament has significantly increased from 21% in 2008 to 35% in 2016, this has not yet translated into fairer conditions for women across the population. For example, levels of gender inequality in rural areas persist despite strides made in advancing the quality of life for women in urban areas [25].

The utilization of a consultative process has also been a cornerstone of the development of gender strategies in the region. For example, Ghana’s National Gender Policy emerged after meetings with various women’s interest groups as well as civil society stakeholders. Zambia’s gender policy was developed after a consultative process with government agencies that used root cause analysis to understand barriers and propose quality access to health services for maternal health as well as sexual and reproductive health outcomes [26].

Prior to the pandemic, Kenya’s national policy on gender and development and South Africa’s national gender policy framework focused on expanding universal access to sexual and reproductive health services. South Africa’s national gender policy framework acknowledged a resource scarcity for women that impacts access to contraceptives, mental health services as well as measures to protect against g (GBV).

The landscape of national gender strategies in the subset of countries that are the focus of this study set the stage for gender-inclusive planning in each setting. However, operationalizing these commitments in health system planning has yet to be fully realized and is further strained under conditions of uncertainty and urgency, as posed by a health emergency.

## Ethical approval

This study reviewed pandemic response plans that were available in the public domain and did not require ethics approval. However, the main study received ethics approval from McMaster University's Human Research Ethics Committee #MREB# 6468/.

## CRediT authorship contribution statement

**Beverley M. Essue:** Funding acquisition, Investigation, Project administration, Resources, Writing – original draft, Writing - review & editing. Writing - developed final draft. Managed submission and revisions. **Lydia Kapiriri:** Funding acquisition, Investigation, Project administration, Resources, Writing – review & editing. **Hodan Mohamud:** Formal analysis, Writing – original draft, Writing – review & editing. **Marcela Claudia Veléz:** Writing – review & editing. **Suzanne Kiwanuka:** Data curation, Funding acquisition, Methodology, Writing – review & editing.

## Declaration of competing interest

The authors declare that they have no known competing financial interests or personal relationships that could have appeared to influence the work reported in this paper.
