# Supplementary material for: Planning with a gender lens: A gender analysis of pandemic preparedness plans from eight countries in Africa
Source: Health Policy Open. 2023 Dec 12;6:100113. doi: 10.1016/j.hpopen.2023.100113 (PMC10809111; doi:10.1016/j.hpopen.2023.100113)
Supplement: Supplementary data 1 [file mmc1.docx]

**Appendix Table 1. Initial and subsequent COVID-19 Response Plans**

| **Country** | **Citations to Initial Plans** | **Citations to Subsequent Plans** |
| --- | --- | --- |
| Ethiopia | Ministry of Health Ethiopia. National Comprehensive COVID-19 Management Handbook. 2020. | Ministry of Health Ethiopia. Ethiopian Health Care Facility COVID-19 Preparedness and Response Protocol. 2020. |
| Ghana | Republic of Ghana, Ministry of Health. Draft National Strategic COVID-19 Response Plan. 2020. | Republic of Ghana Ministry of Health. Ghana COVID-19 Emergency Preparedness and Response Project and Additional Financing: Environmental and Social Management Framework. 2020. |
| Kenya | The Republic of Kenya Ministry of Health. National 2019 Novel Coronavirus Contigency (Readiness and Erly Reponse) Plan. 2020. | The Republic of Kenya Ministry of Health. Kenya COVID-19 Emergency Response Project (P173820)  Stakeholder Engagement Plan (SEP). 2020. |
| Nigeria | Government of Nigeria. National COVID-19 Pandemic Multi-Sectoral Response Plan. 2020. | Government of Nigeria. National COVID-19 Pandemic Multi-Sectoral Response Plan Version #2. 2020. |
| Rwanda | Republic of Rwanda Ministry of Health. Coronavirus Disease 2019, National Preparedness and Response Plan. 2020. | Republic of Rwanda Ministry of Health. Rwanda COVID-19 National Preparedness and Response Plan. 2021. |
| South Africa | Republic of South Africa Department of Health. Preparedness and Response Plan Novel Coronavirus. 2020. | Republic of South Africa Department of Health. COVID-19 Disaster Response Directions Municipalities and Provinces. 2020. |
| Uganda | Republic of Uganda Ministry of Health. Corona Virus Disease - (COVID-19) Preparedness and Response Plan. 2020. | Republic of Uganda Ministry of Health. Corona Virus Disease - (COVID-19) Preparedness and Response Plan Version #2. 2020. |
| Zambia | Republic of Zambia. COVID-19 Multi Sectoral National Contingency and Response Plan. 2020. | N/A |
