# Supplementary material for: Planning with a gender lens: A gender analysis of pandemic preparedness plans from eight countries in Africa
Source: Health Policy Open. 2023 Dec 12;6:100113. doi: 10.1016/j.hpopen.2023.100113 (PMC10809111; doi:10.1016/j.hpopen.2023.100113)
Supplement: Supplementary data 2 [file mmc2.docx]

**Appendix Table 2 (a-h). Summary of findings from initial and subsequent COVID-19 response plans**

**Appendix Table 2a. Gender analysis matrix – COVID-19 response plans from Ethiopia**

| **PANDEMIC RESPONSE DOMAINS** | **GENDER ANALYSIS DOMAINS** | | | |
| --- | --- | --- | --- | --- |
|  | **Access to Training and Resources** | **Distribution of Labour, Practices, Roles** | **Norms, Values, Beliefs** | **Decision-making Power, Autonomy** |
| **Policies, Laws,  Institutions** | - | - | - | Gender composition on COVID-19 response committee: 38%. *Implicit* |
| **Risk of Exposure** | Provide information, instruction and training on occupational safety and health, including a refresher training on infection prevention and control (IPC) as per protocol  Provide adequate IPC and PPE supplies (masks, gloves, goggles/face shield gowns, hand sanitizer, soap and water, cleaning supplies) in sufficient quantity per standard to healthcare or other staff caring for suspected or confirmedCOVID-19 patients, such  that workers do not incur expenses for occupational safety and health requirements  Provide access to mental health and counseling resources to health care  *Note: By providing training, access to necessary protective equipment (e.g., PPE) and support services (e.g., mental health and counseling resources, the response stands to reduce women’s risk of contracting and spreading COVID-19, allows them to safer at work and stands to mitigate negative impacts. Implicit*  Increase home-based service support by appropriately trained, remunerated, and supplied community health workers  *Note: By strengthening the capacity for homecare, which is largely provided by women, this is especially important to support women and girls who are taking care of those who are sick in the home, important here is that those providing homecare are renumerated. Implicit* | - | - | Development of evidence-based guidance on communication strategies and content suitable for various target audiences, including risk communication for communities and interpersonal communication for contact tracing  *Note: By developing communication strategies and content for different audiences, this will help ensure that communities are being reached and stands to reduce the risk and spread of COVID-19, relevant for women and girls and other vulnerable populations who may not access information using usual fora. Implicit* |
| **Response  to Illness/Treatment** | Selected facilities should dedicate an area for COVID-19 case management and isolation  *Note: By having isolation facilities, this will help support HCWs to reduce the risk and spread of COVID-19. Implicit*  Provide staff education about COVID-19 infection control and update polices as  required  Facilities should avail adequate amounts of PPE including medical masks, N95 masks, goggles, soap, and alcohol-based sanitizer  *Note: By educating staff and providing critical resources this stands to reduce their risk and spread of COVID-19, important for women in these roles. Implicit* |  | - | Develop a strategy to detect and manage symptomatic health care workers so that  health workers will not be harmed and affect the system  Emphasize hand and respiratory hygiene and other infection prevention techniques through education, policies, signage, and easy availability of supplies  *Note: By detecting symptomatic health care workers, this will help reduce spread to other HCWs and patients and their families, important as most women are HCWs. Implicit* |
| **Health Systems: Facilities  and Infrastructure** | Determine strategies to maintain services for at-risk patients during outbreak period (e.g., pregnant, dialysis) but unrelated to COVID  Preparedness should include strategies to maintain essential basic routine services for patients during outbreak period (e.g., pregnant, surgery, inpatient service) unrelated to COVID 19 / Develop a plan to facilitate easy ways for medication refills or obstetrician visits  *Note: By continuing health services, this will reduce the impact of COVID-19 on overall health, especially on vulnerable populations, including women and girls (e.g., pregnant women); Explicit* | Develop service restriction plans in case of staff shortages or increased demand (e.g., respiratory care, nutritional support, pharmacy, laboratory, radiology, elective surgeries/procedures)  *Note: By ensuring a plan for staff shortages or increased demand, this will ensure safer working conditions for HCWs and more patients will be able to be seen, important as most women are HCWs*  Develop contingency plans in case of staff shortages or increased demand (e.g.,  respiratory care, nutritional support, pharmacy, laboratory, radiology, elective surgeries/procedures)  Monitoring guide for staff illness and work leave should be available  *Note: By providing staff safe working conditions (e.g., work leave) this will ensure safer working conditions for HCWs and potentially allow more patients will be able to be seen. Implicit* | - | Maintain appropriate working hours with breaks as per the capacity and burden of the facility  Guidelines to support staff to avoid risk of infection or danger to life or health, until the employer has taken any necessary remedial actions  Modify staff responsibilities and shifts as required (supervisory staff work clinically, suspend most education and other administrative burdens), determine where less-trained staff can safely provide support and the extent of family member support  Ensure supervisors are informed of fair staff management plans (e.g. payments, compensatory time off, psychosocial support, etc.).  *Note: By providing staff safe working conditions (e.g., work leave) this will ensure safer working conditions for HCWs and potentially enable more patients will be able to be seen. Implicit* |
| **Economic Impacts** | - | - | - | *-* |
| **Social Impacts** | Implement measures to enable transitions to online school *[Explicit]* | - | - | - |
| **Security Impacts** | - | - | - | The security impacts detailed in the plan did not reflect implicit or explicit considerations of gender. |

The table outlines illustrative segments of verbatim or summarized text extracted from the plan (See Appendix Table 1). The italicized font is the authors’ assessment of the relevance and connection of the text to the matrix domain as either an *Implicit* or *Explicit* consideration of the impact of the response on women and girls on this context. The lighter shade text reflects additions or changes extracted from the subsequent plans.

Implicit: This indicates that while the plans did not explicitly specify that the response was intended to mitigate impacts for women and girls specially, there is a perceived implicit impact (positive or negative). For example, given that women are the majority of health care workers (HCWs), responses that target HCWs stand to benefit women by mitigating the impacts for women in this role.

Explicit: Here women and/or girls are called out specifically as a target for the response.

(-) denotes no explicit or implicit consideration of impacts that might affect women and girls in the COVID-19 response plans reviewed.

**Appendix Table 2b. Gender analysis matrix – COVID-19 response plans from Ghana**

| **PANDEMIC RESPONSE DOMAINS** | **GENDER ANALYSIS DOMAINS** | | | |
| --- | --- | --- | --- | --- |
|  | **Access to Training and Resources** | **Distribution of Labour, Practices, Roles** | **Norms, Values, Beliefs** | **Decision-making Power, Autonomy** |
| **Policies, Laws,  Institutions** | - |  | COVID-19 plan specified an aim to develop a response that reflected and supported progress towards UHC*. Implicit* | COVID-19 response plan developed through a whole-of-government and whole-of-society approach*. Implicit*  Gender composition of COVID-19 planning committee: not reported. *Implicit* |
| **Risk of Exposure** | Train and resource rapid response teams (RRTs) for effective investigation and response  Train health care workers (HCWs) (clinical and non-clinical) on appropriate WASH-IPC practices including all standard precautions, additional precautions, administrative controls, safe burial of Covid-19 deaths and treatment of healthcare waste (both solid and liquid)  Train HCWs in infection, protection and control measures  Provide relevant Personal Protective Equipment and Supplies  Provide psycho-social support to staff and victims/family/community  Provide Social Protection and Psycho-social Support Services to the Vulnerable  Resource mental health hospitals with isolation / quarantine / holding facilities  *Note: Training on RRTs and IPC and resources for protective equipment and other support stands to reduce risk of exposure and infection for HCWs. Access to* psychosocial supports helps to mitigate the negative impact of the pandemic for HCWs. *By orienting communities, which include vulnerable groups (e.g., women) this will reduce their risk of contracting COVID-19. Implicit* | Recruit and train laboratory staff  Strengthen capacity for home care  *Note: Strengthening the laboratory capacity and capacity for homecare, anticipates the workforce burden across the system, especially in roles predominately carried out by women. Implicit*  Conduct community entry programmes to ensure community ownership and participation in all community-based interventions such as identification and assessment of potential isolation and treatment centres  Strengthen community engagement and education activities to ensure adherence to prevention measures using community champions and traditional leaders)  Collaborate with civil society organization and other relevant stakeholders to embark on Stigma Reduction campaigns  Involve traditional and religious leaders in issuing press statements and other media engagements on COVID-19  *Note: By involving communities within the COVID-19 response, this fostered buy-in and stood to improve outreach to those who may not receive information from more formal channels. Implicit* | Provide orientation to communities on appropriate Infection, Prevention and Control Practices  Involve Queen mothers and chiefs in the sensitization of Market women to adhere to COVID-19 protocols and other health issues  *Note: The community-focused campaigns targeted community and faith leaders to broaden the reach of communication about risk of exposure. This can also help to shift norms, values and behaviours to control the risk of exposure. Implicit* | Improve hygiene standards and social distancing in all markets, lorry parks and other places  *Note: Community based hygiene and social distancing policies were relevant for those in informal and precarious work. Implicit*  Coordinate school-based measures, including: use cohort approach to reduce the number of students per class to ease congestion; ensure that all schools have designated holding rooms for suspected COVID-19 cases; support schools to set up isolation / quarantine / holding facilities; disinfect schools (Pre-tertiary and tertiary schools); promote social distancing protocols, washing of hands, wearing of facemasks and other PPEs at the schools  *Note: While community control measures through school closures and other measures supported controlling the spread of the virus and associated impacts for over-burdened health systems and HCWs, it also negatively impacted women, who faced increased care and home-schooling responsibilities, and girls, who had higher school drop-out rates. Implicit* |
| **Response  to Illness/Treatment** | Identify and test suspected cases  Quarantine and test close contacts of confirmed cases  Designate and resource quarantine centres  Designate Isolation and Treatment centres in facilities  *Note: Testing, identification and quarantine measures helped to control the spread of COVID-19, which had relevance for HCW burden and ensuring access to these measures by vulnerable groups in the community. Implicit* | - | - | - |
| **Health Systems: Facilities  and Infrastructure** | - | *-* | *-* | Implement a range of economic stimulus packages and incentive programs:   - Economic Stimulus packages (focused on water and electricity) - Incentives to healthcare workers (including income tax relief on salaries; 50% Basic Salary top up for defined frontline workers) - Reduction in the cost of basic services - Support to businesses and workers - Ensure food security   *Note: The economic stimuli and social assistance programs strengthened the social protections available to buffer the economic ‘shocks’ caused by the pandemic closures. This had relevance for women and girls who were disproportionately negatively impacted by the pandemic (e.g., loss of work/income). Also, by providing incentives to healthcare workers this support fairer renumerated and compensation during the pandemic. Implicit* |
| **Economic Impacts** | - | - | - | *-* |
| **Social Impacts** | - | - | - | - |
| **Security Impacts** | - | - | - | The security impacts detailed in the plan did not reflect implicit or explicit considerations of gender. |

The table outlines illustrative segments of verbatim or summarized text extracted from the plan (See Appendix Table 1). The italicized font is the authors’ assessment of the relevance and connection of the text to the matrix domain as either an *Implicit* or *Explicit* consideration of the impact of the response on women and girls on this context. The lighter shade text reflects additions or changes extracted from the subsequent plans.

Implicit: This indicates that while the plans did not explicitly specify that the response was intended to mitigate impacts for women and girls specially, there is a perceived implicit impact (positive or negative). For example, given that women are the majority of health care workers (HCWs), responses that target HCWs stand to benefit women by mitigating the impacts for women in this role.

Explicit: Here women and/or girls are called out specifically as a target for the response.

(-) denotes no explicit or implicit consideration of impacts that might affect women and girls in the COVID-19 response plans reviewed.

**Appendix Table 2c Gender analysis matrix – COVID-19 response plans from Kenya**

| **PANDEMIC RESPONSE DOMAINS** | **GENDER ANALYSIS DOMAINS** | | | |
| --- | --- | --- | --- | --- |
|  | **Access to training and resources** | **Distribution of labour, practices, roles** | **Norms, values, beliefs** | **Decision-making power, autonomy** |
| **Policies, laws,  institutions** | - | - | - | National COVID-19 task force housed within the Ministry of Health with aligned budget. Called for the protection of vulnerable populations, including women *Explicit*  Gender composition on COVID-19 response committee: 19%. *Implicit* |
| **Risk of Exposure** | Provision on hand washing and disinfection facilities in communities, facilities, and institutions  Availability of disinfectants and antiseptics  *Note: By providing hand washing and disinfection facilities and supplies, including in the community, stands to reduce the risk of transmission and spread of COVID-19. Implicit*  Print and distribute information on infection, exposure and control materials (e.g., posters, brochures, roll up banners, fact sheets)  Publish electronic infection, exposure and control materials through all media outlets  Develop key messages for different audiences (media spokesperson, policy makers, the public, health care workers, CHWs)  *Note: The need to have training and resources on exposure risks was outlined in the plans, they did not specify tailoring the messages to diverse populations. By developing and sharing targeted messaging on risks for different populations, this aimed to reduce the risk of contraction. Implicit* | Engage diverse stakeholders (Religious Leaders & bodies, Health Professionals and traditional leaders) in the communication about risk of exposure.  *Note: Engaging key stakeholders is key to establish trust/buy in from communities. No specific mention of the relevance of this strategy to reach women and girls. Implicit*  Sensitize health workers, including community health workers on 2019-nCoV detection, reporting, investigation, contact tracing, sample collection and shipment  *Note: By sensitizing health workers and community volunteers (which are likely majority women) to exposure risks, this stands to reduce their risk of contracting COVID-19. Implicit*  Train national rapid response teams (RRTs) in 14 selected counties  Train laboratory staff (2 per county) on sample collection, packaging and shipment  *Note: By enhancing training and support for all cadres of health workers ensures that HCWs are trained and supported to contribute to the response. Implicit* | Organize at least one community dialogue session in each community in the 14 high risk counties and among high risk groups  Intensify community mobilization activities for awareness creation/raising, case finding/ reporting and contact tracing  Hold public barazas during preparedness phase  *Note: Holding public dialogue discussions and engagements aimed to reach those who aren't accessing information through other channels. Women and girls are not called out specifically in the groups to be engaged. Implicit*  Continuous behavior assessment and community sensitization through mobile feedback (text messages, social media platforms) and dedicated radio call-in shows using both mainstream and indigenous languages to ensure preventative community and individual health and hygiene practices in line with national public health containment recommendations  Systematically establish community information and feedback mechanisms including through social media monitoring; community perceptions, knowledge, attitude and practice surveys; and direct dialogue and consultations  Ensure changes to community engagement approaches are based on evidence and needs, and ensure all engagement is culturally appropriate and empathetic  *Note: By gathering information on community beliefs and feedback can inform gaps with the current plan and may provide insight into missed gendered impacts of COVID-19 on women and girls. The plan mentions the inclusion of vulnerable groups, in particular women, when engaging with stakeholders. Implicit*  Design, production and distribution of Information Education and Communication (IEC) materials  Publishing electronic IEC materials through all media outlets, including translation of messages into various vernacular languages  Prepare local messages and pre-test through a participatory process, specifically targeting key stakeholders and at-risk groups  Use of two-way ‘channels’ for community and public information sharing such as hotlines (text and talk), responsive social media such as U-Report where available, and radio shows, with systems to detect and rapidly respond to and counter misinformation  *Note: Distributing information on preventative measures can reduce the risk of contraction and spread. There is mention of inclusion of vulnerable groups, in particular women, when engaging with stakeholders. By preparing messages through a participatory process and pre-testing, this helps to ensure that messaging is effectively targeted to the public including at-risk groups. By detecting and responding to misinformation, this stands to reduce the risk of spread and contraction of COVID-19 within communities. Implicit* | Develop National 2019-nCoV contingency plan  Counties to adopt and customize the 2019-nCoV contingency plan  Develop a 2019-nCoV community engagement plan  *Note: If a gender lens is not incorporated within the development of the plan there may be negative impacts on women and girls. Implicit* |
| **Response  to illness/treatment** | - | Training for frontline Workers on case management, IPC and psychosocial support in 14 high risk counties  *Note: By enhancing training and support for all cadres of health workers ensures that HCWs are trained and supported to contribute to the response.*  Train all health workers at all levels of the health system on relevant guidelines and protocols  *Note: Training and support to health workers, ensures that HCWs are trained and supported to contribute to the response. Implicit*  Establish isolation rooms in level 4 health facilities in the 14 high risk counties  Strengthen capacity of Kenyatta National Hospital Infectious Disease Unit Mbagathi, Kenyatta University Teaching and Referral Hospital and Moi Teaching and Referral Hospital to manage infectious diseases – including structural changes to improve negative pressure airflow, floor and air quality, etc  *Note: By strengthening the capacity of hospitals to better manage COVID-19, this will likely better protect HCWs (which are majority women) and/or other patients from contracting and spreading COVID-19. Implicit* | - | - |
| **Health system - facilities  and infrastructure** | Procure relevant Personal Protective Equipment (Gowns, boots, Goggles/Face shields, Gloves, face masks etc.)  WASH supplies  *Note: By providing PPE this will reduce the risk of contraction and spread of COVID-19, this is especially important for HCWs working in public facing roles. Implicit* | Conduct operational research (desk reviews, empirical studies) on risky behaviour pertaining to 2019-nCoV for frontline workers including health workers  *Note: Conducting research on HCWs (which are majority women), may provide insight into gendered impacts of COVID-19 on women HCWs. Implicit* | Rapid community behavior assessment to gather information about different groups knowledge, attitudes, beliefs, and challenges related COVID-19 response  *Note: Gathering information on community beliefs on the COVID-19 response plan can inform gaps with the current plan and may provide insight into missed gendered impacts of COVID-19 on women and girls. Later on in the plan there is mention of inclusion of vulnerable groups, in particular women, when engaging with stakeholders. Explicit* | Document lessons learned to inform future preparedness and response activities  *Note: Support learning from the pandemic response process and opportunities to redress inequities in future preparedness planning. Implicit* |
| **Economic impacts** | - | Allowances for all frontline workers during early response in 14 counties  Allowances, fuel and vehicle maintenance for the RRTs  *Note: Women are the majority of healthcare workers/frontline workers during the pandemic, by providing allowances, this will positively impact women in these roles. Implicit* | - | - |
| **Social impacts** | Implement measures to enable transitions to online school (e.g. materials for distance education; radio schooling) *[Explicit]* | - | - | To reduce vulnerability particularly of women and girls to heinous crimes such as gender-based violence  *Note: By highlighting that women and girls are at an increased risk of GBV during the pandemic is important, but the plan did not outline specific action items to ensure women and girls are better supported. Explicit* |
| **Security impacts** | - | - | - | The security measures detailed in the plans did not reflect implicit or explicit considerations of gender. |

The table outlines illustrative segments of verbatim or summarized text extracted from the plan (See Appendix Table 1). The italicized font is the authors’ assessment of the relevance and connection of the text to the matrix domain as either an *Implicit* or *Explicit* consideration of the impact of the response on women and girls on this context. The lighter shade text reflects additions or changes extracted from the subsequent plans.

Implicit: This indicates that while the plans did not explicitly specify that the response was intended to mitigate impacts for women and girls specially, there is a perceived implicit impact (positive or negative). For example, given that women are the majority of health care workers (HCWs), responses that target HCWs stand to benefit women by mitigating the impacts for women in this role.

Explicit: Here women and/or girls are called out specifically as a target for the response.

(-) denotes no explicit or implicit consideration of impacts that might affect women and girls in the COVID-19 response plans reviewed.

**Appendix Table 2d. Gender analysis matrix – COVID-19 response plans from Nigeria**

| **PANDEMIC RESPONSE DOMAINS** | **GENDER ANALYSIS DOMAINS** | | | |
| --- | --- | --- | --- | --- |
|  | **Access to Training and Resources** | **Distribution of Labour, Practices, Roles** | **Norms, Values, Beliefs** | **Decision-making Power, Autonomy** |
| **Policies, Laws,  Institutions** | National COVID-19 task force housed within the Ministry of Health; stipulations to ensure access to subsistence |  | - | National COVID-19 task force housed within the Ministry of Health with aligned budget. Called for the protection of vulnerable populations, including women. *Explicit*  Gender composition on COVID-19 response committee: 17% *Implicit* |
| **Risk of Exposure** | Prepare educational and training materials on Infection Prevention & Control (IPC) for frontline health workers and first responders  Procure and maintain stockpiles of appropriate medication, vaccines (if applicable), Personal Protective Equipment (PPE) and other consumables   - Issue PPE to appropriate personnel in all sectors - Increase stockage of supplies, PPE and consumables for frontline healthcare workers - Maintain strategic stockpiles of the most important PPE for healthcare workers who may encounter patients with the virus and work out a system to monitor them daily with additional stock ordered where necessary" - Produce and distribute COVID-19 IEC materials including pamphlets, posters, and roll-up banners - Conduct advocacy to the traditional and religious leaders at national, state and LGA level including other community influencers   *Note: By providing PPE, supplies, training and educational materials regarding IPC etc, this stands to address the risk of contracting COVID-19 in health care workers and is thus relevant for women in these roles. Increased supplies to those who may have limited access to PPE/protection (e.g., those in community vs. hospital) will also impact the risk of women contracting COVID. Producing and distributing IEC materials in the community and engaging with traditional and religious leaders build trust and ensures COVID-19/public health messaging is available to a wider audience, including to women and girls who may not receive the messaging from other channels. Implicit*  To address HCW Infection and Poor IP compliance in healthcare facilities:   - "Finalize the COVID-19 healthcare worker safety strategy document" - "Organize online/face to face trainings and webinars on implementation of the healthcare worker safety strategy by engaging professional bodies and associations." - "Finalize National IPC guidelines, engaging all relevant stakeholders and deployment to health facilities" - "Deploy COVID-19 HCW Infection Surveillance and Investigation tool in treatment centers" - "Deploy IPC score card in all health facilities" - "Institute the use of HCW investigation tool for HCW with confirmed COVID 19"   Coordinate the supply of PPEs:   - "Work with Logistics to have access to Infection Surveillance and Investigation tool and track PPE supply" - "Ensure IPC focal persons in treatment centers report weekly PPE stock levels" - "Continuous training (refresher training) on proper use of PPEs through workplace reminders, guidelines"   Implementation of IPC activities at states and health facilities:   - "Institute a facility level IPC programme with a dedicated and trained team or at least an IPC focal point supported by the national, state and facility senior management" - "Train/support health facility IPC focal persons to utilize multimodal strategies to implement 5 key standard precautions for COVID 19 (Hand hygiene, respiratory hygiene, appropriate use of PPE, environmental cleaning and waste management)" - "Engage hospital management, professional bodies and associations on the implementation of the healthcare worker safety strategy" - "Advocate to SMOH and hospital management to own and support IPC activities at state and health facility level" and "Tracking quality of trainings conducted at state and facility level"   *Note: By ensuring better implementation of IPC activities, this will help reduce the risk of HCWs contracting and spreading COVID-19 which is important as women make up the majority of HCWs, as well as communities and vulnerable populations (women and girls). By providing PPE and ensuring an adequate supply of PPE, this will help reduce the risk of HCWs contracting and spreading COVID-19 which is important as women make up the majority of HCWs. Implicit* | - | Low Risk Perception:   - "Review messages being disseminated on COVID 19 and incorporate threat and efficacy element into future messages" - "Consider targeting specific audience rather than generalizing messages" - "Enhance the 'Take Responsibility' campaign to address people across all demographics"   *Note****:*** *By increasing the risk perception of COVID-19 and developing targeted messaging this stands to reduce the spread of COVID-19 as people become more cautious, which is important as women and girls may have not received public health messaging through other channels and targeted messaging may better reach them. Implicit* | - |
| **Response  to Illness/Treatment** | - Provide counselling services for mental and psychosocial issues for health workers (i.e. activate rest and recuperation sites, and confidential telephone support lines)   *Note: women represent the majority of health workers and providing counselling services has relevance for addressing the mental burden/impact of the pandemic*  To address challenges with identifying cases”   - "Enhance Community Based Surveillance for COVID-19 to improve active case handling and sample collection and enhance testing" - "Train community health workers on case findings" - "Conduct routine sample collection outreaches in communities, markets, and other public locations" - "Expand Event Based Surveillance and alert management at LGA, Wards and Communities"   *Note: Enhancing community surveillance helped with reducing the spread of COVID-19 within communities, potentially improving access and opportunities for screening for women and girls (e.g., essential workers, those working in informal sectors, caregivers, etc.). High female labour force participation in community sectors identified. Implicit*  To address challenges with sub-optimal contact tracing and follow-up in sub-national areas:   - "Review the SOP/guidelines for Contact tracing" "Provide Logistics for contact training (people, equipment, PPEs, telephones)" - "Recruit surge capacity for States" - "Conduct gap-specific training to build prerequisite skills among the available State and Local Government Areas" - "Provide guidelines for Psychosocial support and counselling" - "Train and deploy Community informants and volunteers across all LGAs to enhance contact tracing, reporting and follow-up"   *Note: By providing increased training and support, this will help support HCWs who are largely women, and reduce the risk of contracting and spreading of COVID-19 in communities.* | - | - | **INITIAL PLAN**   - Issue pandemic precaution guidelines to emergency departments, hospitals, businesses, airlines, schools/universities, day care facilities, jails/prisons, and other stakeholders, as well as the public   *Note: High female labour force participation in these sectors. Implicit*  To address resistance to isolation decisions   - "Provide supports to states to enforce the case management guidelines for home care" - "Review and revise existing guidelines and algorithms" - "Train staff on psychosocial support" - "Strengthen the mechanisms of observation in isolation centres and screening" - "Conduct risk communication to lower stigmatization"   *Note: Reducing resistance to isolation is important to ensure public health guidelines are followed and risk is minimized, but if social support is not provided (e.g., childcare, financial support), that may also be a barrier to having people follow isolation guidelines, especially for vulnerable populations, such as women. Implicit*  Inadequate treatment and  isolation capacity:   - "Support implementation of home-based care protocol across all state" - "Develop and support the roll out a triaging protocol for proper classification of COVID positive cases" - "Review and improve existing guidelines on isolation of cases including homecare - "Establish community support centres to align with established guidelines"   *Note: By providing adequate treatment and isolation facilities, this will help ensure that the risk of spread for those working in these facilities is minimized. Implicit* |
| **Health Systems: Facilities  and Infrastructure** | - | - | - | - |
| **Economic Impacts** | - | - | - | - Coordinate with the private sector to ensure maintenance of critical civil services (i.e. pharmaceuticals, retail food, retail fuel, etc.)   *Note: given female labour force participation in the retail sector, keeping/maintaining civil/retail services kept women employed* |
| **Social Impacts** | Develop and adjust plans and programs for social protection and humanitarian interventions to target vulnerable populations during the pandemic  Increase stockage of supplies (food and non-food items) and scale up programs and interventions to expand reach to poor and vulnerable groups (i.e., urban areas and remote areas)  Continue to work with welfare, faith-based, and community agencies and groups to provide individuals in need of social protection services  *Note: vulnerable populations may include women and girls and these programs reduce the impact of the pandemic on these populations, plan defines vulnerable populations as: person with disabilities; The unemployed, elderly, IDPs and others. Explicit* | Promptly isolate suspected cases, and provide effective treatment of confirmed cases with improved clinical outcomes, reducing morbidity and mortality  *Note: potential unintended impacts with isolating suspected cases. This may have some gendered differences and impacts (e.g., a woman who has children, may be unable to care for her children and if her partner is isolating that may increase the amount of care work she provides). Implicit* | Assess community willingness to adhere to non-pharmaceutical  interventions (e.g. social distancing) as well as trust for and connectedness to public health agencies for COVID-19, with view to enhancing risk communication and community  engagement strategies  *Note: By assessing the willingness of communities to adhere to non-pharm interventions, this will help evaluate the effectiveness of the plan and how the pandemic is impacting vulnerable communities such as women and girls. Implicit* | Recommend cancellation of large public gatherings and recreation activities and the closure of schools, colleges, universities, and office buildings.  *Note: By closing large gatherings and schools, this had the potential to negatively impact women, as they may be unable to work (e.g., as street vendors) and there is an increased burden for childcare. Implicit*  In phase 6 (recovery) recommended reopening / resuming, which then will have a positive benefits for the women and girls who were negatively impacted by the closures  Reduce the disruption of critical social and economic utilities during COVID-19 Pandemic  *Note: By providing social and financial support (e.g., food, childcare, etc.) this will help reduce the non-health impacts that fall heavily on women and children. Implicit* |
| **Security Impacts** | - | - | - | Plan to collect sex-based data as part of quarantine reporting and for foreign travelers during points of entry  *Note: Sex-disaggregated data supports a better understanding of differences in risk and outcomes. [Explicit]* |

The table outlines illustrative segments of verbatim or summarized text extracted from the plan (See Appendix Table 1). The italicized font is the authors’ assessment of the relevance and connection of the text to the matrix domain as either an *Implicit* or *Explicit* consideration of the impact of the response on women and girls on this context. The lighter shade text reflects additions or changes extracted from the subsequent plans.

Implicit: This indicates that while the plans did not explicitly specify that the response was intended to mitigate impacts for women and girls specially, there is a perceived implicit impact (positive or negative). For example, given that women are the majority of health care workers (HCWs), responses that target HCWs stand to benefit women by mitigating the impacts for women in this role.

Explicit: Here women and/or girls are called out specifically as a target for the response.

(-) denotes no explicit or implicit consideration of impacts that might affect women and girls in the COVID-19 response plans reviewed.

**Appendix Table 2e. Gender analysis matrix – COVID-19 response plans from Rwanda**

| **PANDEMIC RESPONSE DOMAINS** | **GENDER ANALYSIS DOMAINS** | | | |
| --- | --- | --- | --- | --- |
|  | **Access to Training and Resources** | **Distribution of Labour, Practices, Roles** | **Norms, Values, Beliefs** | **Decision-making Power, Autonomy** |
| **Policies, Laws,  Institutions** | - | - | Development of response stipulated the need for initial capacity assessment and risk analysis, including mapping of vulnerable populations. | Gender composition of COVID-19 planning committee: not reported. *Implicit* |
| **Risk of Exposure** | Ensure access to hand hygiene/WASH services in public places and community spaces most at risk  *Note: By providing access to hand hygiene/WASH services in public places and community spaces most at risk, this will reduce the spread of COVID-19 in vulnerable communities, which include women and girls. Implicit*  Conduct trainings on COVID-19 case identification, triage, reporting, contact tracing, infection prevention and control, etc. to health facility staff  Train frontline healthcare workers on sample collection for screening and surveillance activities  *Note: By providing training to HCWs, which are mostly women, this will likely reduce their risk of contracting and spreading COVID-19 and being safer at work. Implicit*   Procure and distribute COVID-19 specific medical and non-medical supplies, including personal protective equipment  *Note: By providing COVID-19 medical and non-medical supplies, this will help reduce the risk of contraction and spread of COVID-19 for HCWs, especially important for HCWs who are women. Implicit* | Train health promotion officers at sub-national levels  *Note: Training health promotion officers stands to reduce their risk of contracting and spreading COVID-19. Implicit* | Establish community information and feedback mechanisms  *Note: By establishing community information and feedback mechanisms this will help buy-in and trust from communities, which is especially important for women and girls who may not receive public health messaging otherwise/from other channels. Implicit* | - |
| **Response  to Illness/Treatment** | Strengthen COVID-19 data management systems  Promote COVID-19 data use  *Note: By encouraging data collection and use, this will help inform planning and implementation. However, the plan did not indicate disaggregating data by sex/gender.*  Strengthen home based care program  *Note: By strengthening the capacity for homecare, this is important to support women and girls who are taking care of those who are sick in the home*  Ensure adequate financial support and trained human resources for vaccine supply, delivery, monitoring, and evaluation  *Note: Women comprise most healthcare workers and will likely be delivering vaccines, so by providing support, this will help reduce their risk of adverse events at work* |  | Development of response stipulated the need for initial capacity assessment and risk analysis, including mapping of vulnerable populations.  Note: This set the stage for inclusion of the perspectives of made-vulnerable populations in the development of the response. Implicit. | Guidelines to identify and monitor COVID-19 infections in high-risk groups including contacts of confirmed cases  Guidelines to monitor COVID-19 associated deaths in communities and health facilities  *Note: By identifying and monitoring infections and deaths in communities, this will help provide communities and government with a better understanding of who is being impacted and who is being supported by the current plan, which will help inform planning and ideally reduce the risk and spread of COVID-19 in communities, especially for women and girls. Implicit*  Monitor the quality of COVID-19 surveillance data and interventions  Strengthen monitoring and evaluation of COVID-19 immunization program implementation and evaluate COVID-19 vaccine introduction, impact and effectiveness  *Note: By monitoring surveillance data and interventions, this will help inform planning and ideally reduce the risk and spread of COVID-19 in communities, especially for women and girls. Implicit*  Support care of the most vulnerable groups and key populations (e.g., prisoners, refugees, the elderly, those who comorbidities, children in care institutions, etc.)  *Note: Defined vulnerable groups and key populations and stipulated the resources and support to be made available, stands to reduce the risk, spread and negative outcomes of COVID-19 for these populations. Explicit*  Define, identify, and estimate the size of target populations and vaccination delivery strategies  *Note: By prioritizing healthcare workers, other frontline workers, and other vulnerable populations (e.g., elderly), this will reduce their risk of serious illness, hospitalization, and death due to COVID-19. Implicit* |
| **Health Systems: Facilities  and Infrastructure** | Ensure access to hand hygiene/WASH services in public places and community spaces most at risk  *Note: By providing access to hand hygiene/WASH services in public places and community spaces most at risk, this will reduce the spread of COVID-19 in vulnerable communities, which include women and girls. Implicit*  Conduct supportive supervision on COVID-19 to health facility staff  Conduct trainings on COVID-19 case identification, triage, reporting, contact tracing, infection prevention and control, etc. to health facility staff  Train frontline healthcare workers on sample collection for screening and surveillance activities  *Note: By providing training and support to health facility staff, this will support safer working conditions for HCWs. Implicit*  Procure and distribute COVID-19 specific medical and non-medical supplies, including PPE  *Note: By providing COVID-19 medical and non-medical supplies, this will help reduce the risk of contraction and spread of COVID-19 for HCWs. Implicit*  Strengthen governance and coordination mechanisms for continuity of essential health and nutrition services, re-organize and optimize service delivery platforms to ensure uninterrupted provision of essential health services in the context of COVID-19  *Note: By continuing to provide essential health services, this will reduce the impact of the pandemic on especially vulnerable populations, including women and girls (e.g., GBV support, perinatal support, etc.). Implicit* | Train health promotion officers at sub-national levels  *Note: Training health promotion officers, which are most likely women, this will provide them with more safety at work and reduce their risk of contracting and spreading COVID-19. Implicit* | Establish community information and feedback mechanisms  *Note: Establishing community information and feedback mechanisms this will foster buy-in and trust from communities, which is especially important for women and girls who may not receive public health messaging otherwise/from other channels. Implicit.* | - |
| **Economic Impacts** | - | - | - | Provide support to individuals, households, and communities, especially most vulnerable, to prevent and control COVID-19 through adopting and sustaining key recommended behaviours  *Note: By providing support to communities, this will reduce the impact of COVID-19 on communities, especially important for women and girls, and support efforts to mitigate the economic shock to household and the economy. Implicit* |
| **Social Impacts** | Implement measures to enable transitions to online school (e.g., radio schooling) *[Explicit]* | *-* | Develop strategies for risk communication, community engagement and crisis communication  *Note: By engaging communities and developing strategies for risk communication, this will help with buy-in and ensure information is reaching those who may not receive it through other channels, especially important for women and girls. Implicit* | Enforce self-quarantine of COVID-19 at-risk persons  *Note: By enforcing self-quarantine without social supports (e.g., financial support for missed wages/work, childcare, etc.) this had the potential to negatively impact women and girls. Implicit*  Develop National risk-communication and community engagement plan for COVID-19  *Note: A national risk communication and community engagement plan had the potential to ensure women and girls were reached and engaged. Implicit*  Document lessons learned to inform future preparedness and response activities  *Note: Enables learning that can be updated in future plans. Implicit* |
| **Security Impacts** | - | - | - | The security measures detailed in the plan did not reflect implicit or explicit considerations of gender. |

The table outlines illustrative segments of verbatim or summarized text extracted from the plan (See Appendix Table 1). The italicized font is the authors’ assessment of the relevance and connection of the text to the matrix domain as either an *Implicit* or *Explicit* consideration of the impact of the response on women and girls on this context. The lighter shade text reflects additions or changes extracted from the subsequent plans.

Implicit: This indicates that while the plans did not explicitly specify that the response was intended to mitigate impacts for women and girls specially, there is a perceived implicit impact (positive or negative). For example, given that women are the majority of health care workers (HCWs), responses that target HCWs stand to benefit women by mitigating the impacts for women in this role.

Explicit: Here women and/or girls are called out specifically as a target for the response.

(-) denotes no explicit or implicit consideration of impacts that might affect women and girls in the COVID-19 response plans reviewed.

.

**Appendix Table 2f. Gender analysis matrix – COVID-19 response plans from South Africa**

| **PANDEMIC RESPONSE DOMAINS** | **GENDER ANALYSIS DOMAINS** | | | |
| --- | --- | --- | --- | --- |
|  | **Access to Training and Resources** | **Distribution of Labour, Practices, Roles** | **Norms, Values, Beliefs** | **Decision-making Power, Autonomy** |
| **Policies, Laws,  Institutions** | - | - | - | Gender composition of COVID-19 planning committee: 38%. *Implicit* |
| **Risk of Exposure** | Conduct hygiene promotion campaigns for enhancing IPC practices at facilities and the public  Provide adequate hand washing facilities i.e. Alcohol Based Hand Run (ABHR) or soap and water to health care workers  *Note: Reduce the risk of contraction and spread within women and girls both in public sector (HCWs) and communities*  *Note: Ensure that the health care system is prepared to receive, manage, and report on the clinical progress of persons with COVID-19 in such a way as to minimise the risk to health care workers and maximise good patient outcomes. Implicit*  Provide appropriate PPE and the required equipment including thermal scanners and PPEs  *Note: Limit* contraction and spread of the virus in HCWs*. Implicit*  Adapt messages and produce various infection, exposure and control materials including flyers, banners, posters to inform the public on the Public Health risks of Coronavirus (COVID-19) and the related mitigation measures in various settings through (RCCE)  *Note: By producing IEC materials this will ensure more people are informed about risks and reduce their risk of contracting COVID-19, which is especially important for women and girls working in the public sector (e.g., retail or care workers). No mention of plan to develop tailored communication strategies. . Implicit*  Water and sanitation (available at public facilities, public transport points, services in high population density settlement)  Hygiene education (share awareness, prevention comms, provide staff prevention equipment, with health, other orgs train community leaders as health promoters)  *Note: By providing sanitation and water, this will help reduce the risk of contracting and spreading COVID-19, which is especially important for women and girls both in public sector (retail) and communities. Implicit* | Develop and disseminate guidelines and FAQ documents on infection, protection, and control for all cadres of workers and for the public (Ensure that the health care system is prepared to receive, manage, and report on the clinical progress of persons with COVID-19 in such a way as to minimise the risk to health care workers and maximise good patient outcomes)  *Note: By disseminating IPC documents to workers and the public, this will help reduce their risk of contracting and spreading COVID-19, especially important for women whose roles may put them at higher risk (e.g., HCWs and women working in retail/service roles). Implicit*  Employee Health and Safety (Provide frontline staff with protective equipment. Prioritise electronic document handling. Training. Sanitise/disinfect. Mechanisms for reporting of identified cases. Ensure sufficient stock)  *Note: By prioritizing employee health and safety this will help protect employees and reduce their risk of contracting / spreading COVID-19 which is particularly important for HCWs. Implicit* | Disseminate messages through print media, radio and TV talk shows and the related mitigation measures in various settings through (RCCE)  *Note: Engaging the media in the risk communication will broaden the reach of the risk of exposure messaging. Implicit*  Conduct house to house visits/sensitization in high risk settings (Continuously inform the public on the Public Health risks of Coronavirus (COVID-19) and the related mitigation measures in various settings through (RCCE))  Disseminate IEC messages during public events, confined environments (schools and prisons), Mass Gathering, etc.  *Note: Broaden the reach of the risk communication messaging; also fosters buy-in among the public. Implicit* | Guidelines on enhanced waste management, extra-ordinary public facilities cleansing, identify hotspot areas, mitigation measures, response teams. Share info with local, provincial and national structures.  *Note: Aim to reduce the risk of contracting and spreading COVID-19, which is especially important for women and girls both in public sector (retail) and communities. Implicit* |
| **Response  to Illness/Treatment** | - | - | - | Institutional - Establish Provincial Command Council, coordinating structures at provincial level. Joint operation centres per district/metros. Avail resources. Monitor impact)  *Note: There is no mention of having women being represented or included within the decision making, I wonder if there aren't women represented during decision making if it will lead to missed opportunity for addressing the gendered impacts of COVID-19. Implicit*  COVID-19 Response Plans (Develop, implement Response Plan for province immediately. Monitor, coordinate, report. Analyse district/Metros risk profiles. Support district disaster management centres)  *Note: There is no mention of having women being represented or included within the decision making, I wonder if there aren't women represented during decision making if it will lead to missed opportunity for addressing the gendered impacts of COVID-19.* |
| **Health Systems: Facilities  and Infrastructure** | Establish and maintain screening capacity at key points of entry of health facilities. Ensure that the health care system is prepared to receive, manage and report on the clinical progress of persons with COVID-19 in such a way as to minimise the risk to health care workers and maximise good patient outcomes)  Note: *Reduce the risk of contraction and spread within women and girls both in public sector (HCWs) and communities*  Set up of hotlines for the public and health teams to report suspected cases or rumours (Establish multisectoral coordination at both national and subnational levels for strategic discussions)  *Note: There may be gendered differences in terms of access to phones, impacting access to information and reporting of suspected cases. Implicit* | - | - | - |
| **Economic Impacts** | - | - | - | - |
| **Social Impacts** | Implement measures to enable transitions to online school (e.g. materials for distance education; radio schooling) *[Explicit]* | *-* | *-* | Create a technical expert IPC group and meet at appropriate intervals (Ensure that the health care system is prepared to receive, manage and report on the clinical progress of persons with COVID-19 in such a way as to minimise the risk to health care workers and maximise good patient outcomes)  Create a group of expert clinicians and meet at appropriate intervals (Ensure that the health care system is prepared to receive, manage and report on the clinical progress of persons with COVID-19 in such a way as to minimise the risk to health care workers and maximise good patient outcomes)  Set up regular meetings with provincial communicable disease co-ordinators and other appropriate provincial representatives (Ensure that the health care system is prepared to receive, manage and report on the clinical progress of persons with COVID-19 in such a way as to minimise the risk to health care workers and maximise good patient outcomes)  *Note: If women are not represented in this group / discussions, they decisions may not incorporate a gender lens, leading to missed opportunities to develop planning inclusive of women and girls needs*  Mandatory closures of all municipal spaces (close all non-essential services, public spaces, facilities/pools, beaches, libraries, halls/recreation centres, museums/art galleries, markets, events) and social gatherings/celebrations  *Note: By closing non-essential services, this will negatively impact women, girls and families that rely/use these services, as well as women and girls working in these spaces.*  Isolation/quarantine (identify quarantine, isolation sites with provincial departments and Health Authorities. Implement provisions guided by the protocols. Monitor/enforce measures to combat pandemic.  *Note: By quarantining people without providing social support (e.g. financial support for loss of income or child care if their caregivers are within an isolation facility), this will disproportionately impact women and girls*  Municipal ops/governance (perform various legislated functions, including passing of budgets and adoption of IDPs. Comply with laws prescribing the IDP and budget processes aligned with the COVID-19 regulations)  *Note: There is no mention of having women being represented or included within the decision making.* |
| **Security Impacts** | - | - | - | The security measures detailed in the plan did not reflect implicit or explicit considerations of gender. |

The table outlines illustrative segments of verbatim or summarized text extracted from the plan (See Appendix Table 1). The italicized font is the authors’ assessment of the relevance and connection of the text to the matrix domain as either an *Implicit* or *Explicit* consideration of the impact of the response on women and girls on this context. The lighter shade text reflects additions or changes extracted from the subsequent plans.

Implicit: This indicates that while the plans did not explicitly specify that the response was intended to mitigate impacts for women and girls specially, there is a perceived implicit impact (positive or negative). For example, given that women are the majority of health care workers (HCWs), responses that target HCWs stand to benefit women by mitigating the impacts for women in this role.

Explicit: Here women and/or girls are called out specifically as a target for the response.

(-) denotes no explicit or implicit consideration of impacts that might affect women and girls in the COVID-19 response plans reviewed.

**Appendix Table 2g. Gender analysis matrix – COVID-19 response plans from Uganda**

| **PANDEMIC RESPONSE DOMAINS** | **GENDER ANALYSIS DOMAINS** | | | |
| --- | --- | --- | --- | --- |
|  | **Access to Training and Resources** | **Distribution of Labour, Practices, Roles** | **Norms, Values, Beliefs** | **Decision-making Power, Autonomy** |
| **Policies, Laws,  Institutions** | - | - | - | Gender composition of COVID-19 planning committee: 20%. *Implicit* |
| **Risk of Exposure** | *-* | Build capacity of laboratory and selected health staff on appropriate COVID-19 sample collection, packaging, handling and transportation  *Note: Support for range of HCWs to engage effectively and safely in the response. Implicit* | -- |  |
| **Response  to Illness/Treatment** | - | Facilitate self-quarantine in the community and homes  *Note: Established self-quarantine requirements but lacked details on the social supports (e.g., financial support for loss of income, support for childcare) available to support individuals to remain at home and in quarantine for extended periods of time. Implicit* | Strengthen community engagement  *Note: Supports inclusion of diverse perspectives in the response and fosters buy-in. Implicit* | "Strengthen community engagement and social protection structures for COVID-19 in Communities"  *Note: Aims to mitigate the impact of the ‘shocks’ caused by COVID to the economy. Implicit*  Conduct a rapid assessment of the Communities in terms of organization; health behaviour and social protection measures; and occupational health and safety in the context of COVID-19  *Note: This assessment stands to adjust the response to account for the potential for different needs within the population. Implicit* |
| **Health Systems: Facilities  and Infrastructure** | Establish COVID-19 quarantine facilities and Facilitate self-quarantine and continuation of essential services for COVID-19 in the community and homes  *Note: By establishing quarantine facilities without providing social supports (e.g., financial support for loss of income, support for childcare), this stands to negatively affect women and girls with an increased care burden.*  Enhance capacity for delivery of essential and emergency medical services  Strengthen required health infrastructure for the response to COVID-19  Ensure proper nutrition and welfare of patients and health care workers managing COVID-19 patients  *Note: By strengthening the health infrastructure this will help reduce the burden on the healthcare system to better provide adequate care to all and HCWs. Implicit*  Build capacity for Mental Health and Psychosocial Support (MHSSP) for COVID-19 effects  *Note: By providing mental health support, this will help reduce the negative mental health impacts of COVID-19. Implicit* | Re-organize and maintain access to essential quality health services  *Note: By continuing essential services, this stands to minimize the health and non-health impacts of COVID-19 and mitigate a worsening of other health conditions. Implicit* | - | - |
| **Economic Impacts** | Provide tailored economic  strengthening, and enhanced child protection through Sustainable Outcomes for Children and Youth (SOCY) project  *Note: social protection measures aim to mitigate the health and non-health impacts of the pandemic if well targeted to those in greatest need. Implicit* | **-** | - | Establish capital, economic recovery (for IGAs) and health packages to the survivors of GBV in order to help re-establish pre-pandemic sources of income and access basic health care esp. those young and expectant  mothers, breast feeding mothers, mothers who have been forced out of their “marital homes”, PLWHA  and SRH and Rights related challenges  *Note: by providing capital/financial support and health packages to those who experienced GBV, this will help mitigate the impacts of GBV during COVID-19 but not focus on prevention of GBV in the response. Implicit* |
| **Social Impacts** | Support integration of social mobilization and community engagement with community-based surveillance and psychosocial support services including refugee settlements  *Note: By providing social supports, this will help reduce the social impacts of COVID-19 on the most vulnerable, which includes women and children among other groups. Explicit*  Provide timely prevention and response to the identified needs in gender-based violence (GBV), violence against children (VAC), Emergency Alternative Care and Children in detention during and post COVID-19  *Note: By providing support for GBV, VAC and emergency alternative care, this will help better address some of the social impacts of COVID-19 faced by women and girls (e.g., GBV and VAC). Explicit*  Support implementation of social protection interventions for vulnerable populations  *Note: By implementing social protection interventions, this will help address some of the social impacts of COVID-19 faced by women and girls (e.g., loss of work/income, increased care work, etc.). Implicit*  Provide modified nutritional support and emergency support services for OVCs identified, tailored economic  strengthening, and enhanced child protection through Sustainable Outcomes for Children and Youth (SOCY) project  *Note: By providing social supports, such as nutritional support and emergency support services for orphans and vulnerable children, this will help reduce the social impacts of COVID-19 on the girls impacted. Implicit*  Implement measures to enable transitions to online school (e.g. radio schooling) *[Explicit]* | *-* Described a role for the heads of households to report COVID-19 exposures directly to the government.  *Note: This had the potential to reduce the agency of women to report on their own health status and at the same time, marginalized households with single females. [Implicit]* | ***-*** | Develop a Response Plan and strategy for GBV/VAC related to COVID-19  *Note: By providing support for GBV, VAC and emergency alternative care, this will help better address some of the social impacts of COVID-19 faced by women and girls (e.g., GBV and VAC) but not focus on prevention of GBV in the response. Explicit*  Strengthen social protection structures for COVID-19 in Communities  *Note: Stands to mitigate the non-health (e.g. social and economic) impacts of COVID-19. Implicit*  Conduct a rapid assessment of COVID 19 on GBV, VAC,  Emergency Alternative care and children in detention,  to inform development of an evidence based prevention  and Response Plan and policy briefs on GBV/VAC,  Emergency Alternative care during crises and emergencies  *Note: This aims to inform the allocation of further resources. Implicit* |
| **Security Impacts** | - | - | - | The security measures detailed in the plan did not reflect implicit or explicit considerations of gender. |

The table outlines illustrative segments of verbatim or summarized text extracted from the plan (See Appendix Table 1). The italicized font is the authors’ assessment of the relevance and connection of the text to the matrix domain as either an *Implicit* or *Explicit* consideration of the impact of the response on women and girls on this context. The lighter shade text reflects additions or changes extracted from the subsequent plans.

Implicit: This indicates that while the plans did not explicitly specify that the response was intended to mitigate impacts for women and girls specially, there is a perceived implicit impact (positive or negative). For example, given that women are the majority of health care workers (HCWs), responses that target HCWs stand to benefit women by mitigating the impacts for women in this role.

Explicit: Here women and/or girls are called out specifically as a target for the response.

(-) denotes no explicit or implicit consideration of impacts that might affect women and girls in the COVID-19 response plans reviewed.

**Appendix Table 2h. Gender analysis matrix – COVID-19 response plans from Zambia**

| **PANDEMIC RESPONSE DOMAINS** | **GENDER ANALYSIS DOMAINS** | | | |
| --- | --- | --- | --- | --- |
|  | **Access to Training and Resources** | **Distribution of Labour, Practices, Roles** | **Norms, Values, Beliefs** | **Decision-making Power, Autonomy** |
| **Policies, Laws,  Institutions** | - |  | - | National COVID-19 task force housed within the Ministry of Health.  Gender composition of COVID-19 planning committee: not reported. *Implicit* |
| **Risk of Exposure** | Strengthen School-WASH for the prevention of COVID-19 transmission at school  Strengthening of WASH and infection prevention and control (IPC) measures in the health facilities/isolation/treatment centres and public places  Improvement of water supply to vulnerable communities, especially in the high-density urban areas and refugee settlements  *Note: Manages the risk of exposure and transmission of COVID at schools, especially important for girls, who will be more negatively impacted by missing school, in health facilities, especially important for HCWs and those living in areas of high burden of COVID-19, and communities, especially important for those living in areas of high burden of COVID-19. Implicit* | Train and equip rapid-response teams to investigate cases and clusters early in the outbreak, and conduct contact tracing within 24 hours  Support training of laboratory personnel on COVID-19, specimen packaging, transportation, biosafety and biosecurity  Disseminate and train various technical healthcare and community care workers guidelines and protocols for management of severe acute respiratory infections and COVID-19  *Note: Emphasis on training a range of different individuals supporting the COVID-19 response manages their own risk of exposure and also broadens the reach of the efforts to control exposure. Implicit* | Implement national risk-communication and community engagement plan for COVID-19, including details of anticipated public health measures  *Note: Developing a community engagement plan is key for buy-in for communities and to ensure messaging is communicated effectively, the plan has a focus on groups such as women and girls, so if messaging is adapted to them, they may better be able to inform more women and girls of public health messaging. Implicit* | Delivery/dissemination of key information and messages on COVID-19 prevention through schools and educational institutions  *Note: Signals cross-sectoral collaboration and alternate platforms used for risk and communication; relevance for reaching communities, ability to keep schools open and education outcomes for girls. Implicit* |
| **Response  to Illness/Treatment** | Map vulnerable populations (including, but not limited to those with chronic diseases, the elderly, women and children, including migrants, refugees, internally displaced persons, populations in detention) and public and private health facilities (including traditional healers, pharmacies and other providers) and identify alternative facilities that may be used to provide treatment  *Note: By mapping populations (such as women and girls, among other groups) alongside available facilities, this stands to ensure efforts /resources are in place to address the needs of these groups*  Provide technical and operational support through short to medium term secondment of staff; and provide life-saving primary health care and procurement of critical medicines and medical supplies, support to infrastructure, especially in humanitarian settings  *Note: By providing support and access to supplies, this will help ensure that HCWs are able to treat more patients and carry out their work more safely (which is important as most HCWs are women) . Implicit* | Provide technical and operational support through short to medium term secondment of staff  *Note: Deployment of additional HCWs from elsewhere in the system addressed the workload challenge of frontline workers but also created pressures elsewhere in the system. Also potential for unintended consequences from change in role / increase in workload for redeployed staff. . Implicit*  Disseminate infection, protection and control (IPC) guidance for home and community care providers  *Note: By providing IPC guidance to home and community care providers, which are majority women, this will help reduce the risk and spread of COVID-19, which is especially important as home and community care providers typically are not prioritized in comparison to HCWs working in hospital. Implicit* | - | - |
| **Health Systems: Facilities  and Infrastructure** | Set up COVID-19 designated isolation facilities in health facilities  *Note: By setting up isolation facilities this will help reduce the spread of COVID-19, which is important for HCWs and vulnerable groups (e.g., women) . Implicit*  Support continuity of SRH, HIV/GBV service provision, such as Safe Delivery services, Family Planning, Postnatal STI and HIV treatment and SGBV services  Ensure that the emergency maternal referral system is maintained to prevent loss of lives while giving birth  Work with community structures to ensure community based services are available including information and SRH commodities especially for pregnant women and girls who need to frequently visit health facilities  *Note: Continuity of essential care is key to mitigate a worsening of health outcomes for women and girls. Explicit*  Provide menstrual hygiene products to vulnerable women and girls infected with COVID19  *Note: Given economic shock caused by COVID-19, this ensures access to essential supplies for women and girls. Explicit* | Engage and support Community Health volunteers and commodity distributors by ensuring that they are oriented in the prevention of the COVID 19 and ensure that they have adequate PPEs and supplies  *Note: Recognizes the critical role that CHWs have in the COVID-19 response and supports efforts to mitigate risk of infection. Implicit*  Develop and implement a plan for monitoring of healthcare personnel exposed to confirmed cases of COVID‑19 for respiratory illness  *Note: By developing a plan for monitoring exposed HCWs this will ideally reduce the risk of spread to COVID-19 to other HCWs, especially important as women make up the majority of HCWs. Implicit*  Record, report, and investigate all cases of healthcare-associated infections  *Note: By recording and investigating infected HCWs this will ideally reduce the risk of spread to COVID-19 to other HCWs, especially important as women make up the majority of HCWs. Implicit*   Prepare staff surge capacity and deployment mechanisms; health advisories (guidelines and SOPs); pre- and post-deployment package (briefings, recommended / mandatory vaccinations, enhanced medical travel kits, psychosocial and psychological support, including peer support groups) to ensure staff well-being  *Note: By providing guidelines and additional supports including psychosocial support, this will support HCWs (majority women) to do their jobs safely and reduce their risk of adverse events. Implicit*   Support Health System Strengthening by ensuring adequate staffing levels in key facilities to provide routine health services including emergency obstetric services by ensuring HCW have required infection prevention equipment and MISP delivery skills  *Note: By continuing to provide health services including emergency obstetric services this will ensure negative impacts of COVID-19 on other health concerns (outside of COVID-19) are addressed [Explicit] and by providing HCW training, this will support them to adequately do their jobs. Implicit* | - | Develop a national plan to manage PPE supply (stockpile, distribution) and to identify IPC surge capacity (numbers and competence)  *Note: By monitoring and managing PPE this could reduce the risk of contraction and spread of COVID-19 for HCWs, especially important for HCWs who are women. Implicit*  Create data bases and ensure disaggregated data is available to tease out critical elements such as hotspots and most vulnerable population groups in case the evolution of the pandemic will be different in the Zambian context  *Note: Reviewing disaggregated data will help inform the impacts of the plan and pandemic on vulnerable groups (e.g., women and girls) and facilitate the ability to adjust planning if not effective/negatively impacting women and girls. Implicit*  Document lessons learned to inform future preparedness and response activities as well as the production of storytelling archive of pictures, stories and narratives of all steps of the outbreak  *Note: Documenting lessons learned, can inform future pandemic preparedness planning. Implicit*   Disseminate Infection Protection and Control guidance for home and community care providers  *Note: Recognizes the critical role of a range of providers involved in supporting the COVID-19 response. Implicit* |
| **Economic Impacts** | Engage with local donors and existing programmes to mobilize/allocate resources and capacities to implement operational plan  *Note: The mobilization of resources will assist vulnerable populations, which includes women and girls (among other groups) better deal with the negative impacts of the pandemic (e.g., economic impacts). Implicit*  Provision of food and non-food items to target households during shut down  Distribution of essential food and non-food assistance including social cash transfer ahead of lockdown  Ensure emergency food needs are met; adjust social protection programmes; scale up nutritional support; support management and prevention of undernourishment  *Note: By providing food and non-food items, this will help reduce the negative social impacts of COVID-19 on affected families. especially women and girls who may be disproportionately negatively impacted by the pandemic (e.g., loss of work/income). This can also fit under economic impacts. Implicit* | Set-up of mobile money payment system for salary/incentive/support schemes to COVID-19 health-workers and administrative specific surge team  *Note: By providing additional support to HCWs (where women make up the majority of healthcare workers/frontline workers during the pandemic), this will potentially mitigate negative economic impacts for women in these roles. Implicit* | - | Increase cash transfer amounts to recipients of social assistance through a one-off payment (or ensure multiple payments to help families meet their basic needs; providing complementary entitlement to offset loss of income for small-scale producers  *Note: Financial assistance aimed to reduce the negative economic impacts of COVID-19 on affected families, especially relevant for women and girls who may be disproportionately negatively impacted by the pandemic (e.g., loss of work/income). Implicit*  Identify and agree on critical measures that need to be in place to ensure safety of the people working in establishments and shops open to ensure continuity of essential livelihoods and supplies  *Note: By planning for continuity of essential livelihoods and supplies, this will help reduce the negative economic impacts of COVID-19, which is important for women working in the informal economy. Implicit* |
| **Social Impacts** | Prepare for the opening of schools with psycho-social support and accelerated/catch-up learning  *Note: By providing psycho-social support and accelerated/catch-up learning this will help ensure the negative impacts of COVID-19 on education are minimized, which is especially important for girls. Implicit* Ensure social protection facilitates access to health care by reducing out of pocket payments for patients or expanding health insurance coverage and benefits; provides income support where family members are no longer able to work; and prevents a wide range of negative outcomes among children, ranging from malnutrition and sickness to violence and depression  *Note: By providing social assistance to access healthcare and other services, this will help reduce the negative impacts on health (outside of COVID-19 specific related health concerns), which is important for girls and women. Implicit*  Ensure resources to address the increased risks of exposure to gender-based violence to varying degrees  Implement measures to enable transitions to online school (e.g. materials for distance education;) *[Explicit]* | Safeguard the social welfare workforce at district level so as to ensure that statutory services for addressing gender-based violence, violence against children, children without parental care and juvenile offending can continue to operate. Support the workforce protective equipment, with regular high-quality information, advice and counselling  *Note: By ensuring that services for gender-based violence and violence against children and children without parental care and juvenile offenders continue, this will help reduce negative social impacts of COVID-19 and women and children. Explicit* | Community engagement for prevention of violence, discrimination, marginalization, and xenophobia through promotion of social cohesion messaging and activities  *Note: By engaging communities this stands to reduce the negative non-virus related impacts of the pandemic (e.g., gender-based violence). Explicit* | Create databases and ensure disaggregated data is available to tease out critical elements such as hotspots and most vulnerable population groups in case the evolution of the pandemic will be different in the Zambian context  Document lessons learned to inform future preparedness and response activities as well as the production of storytelling archive of pictures, stories and narratives of all steps of the outbreak  *Note: Reviewing disaggregated data will help inform the impacts of the plan and pandemic on vulnerable groups (e.g., women and girls) and facilitate the ability to adjust planning if not effective/negatively impacting women and girls. Also fosters learning that is relevant for future pandemic response planning. Implicit*  Children and affected individuals will be provided with appropriate protection support. Partners will ensure COVID-19 response will not aggravate existing vulnerability and protection risk though active participation of community and provision of appropriate protection support  *Note: By ensuring children are provided with protection support this will help reduce the negative social impacts of COVID-19 on children and affected individuals. Explicit*  COVID -19 response public health activities will be complemented by social, economic and mental health interventions which address important issues that have impact on mental health of affected population involved health workers, survivors, and other actors  *Note: Critical for mitigating the potential negative social/economic impacts of COVID-19 especially on vulnerable groups (e.g., women and girls). Implicit*  Provide technical support to key government ministries and NGOs responding to the pandemic to prioritise GBV prevention, clinical management and referral systems and ensure functionality. Provide mandatory guidance for preventing and responding to COVID in childcare facilities and reformatory schools. Provide support and oversight on guidance implementation  *Note: By ensuring that gender-based violence prevention is prioritized and providing guidance to childcare facilities and schools, this will help reduce negative social impacts of COVID-19 and women and children. Explicit*  Develop and support implementation of Standard Operating Procedures for referrals from health centres to social welfare related to temporary care of children whose parents/cares have been affected  *Note: By providing support for children of caregivers that have been affected by COVID-19, this will help reduce the negative social impacts of COVID-19 on affected families. Explicit*  Provision of Distance Education to all learners, especially the disadvantaged, staying at home due to school closure  *Note: By providing accessible/distance education this will help ensure the negative impacts of COVID-19 on education are minimized, which is especially important for girls. Implicit* |
| **Security Impacts** | - | - | - | The security measures detailed in the plan did not reflect implicit or explicit considerations of gender. |

The table outlines illustrative segments of verbatim or summarized text extracted from the plan (See Appendix Table 1). The italicized font is the authors’ assessment of the relevance and connection of the text to the matrix domain as either an *Implicit* or *Explicit* consideration of the impact of the response on women and girls on this context. The lighter shade text reflects additions or changes extracted from the subsequent plans.

Implicit: This indicates that while the plans did not explicitly specify that the response was intended to mitigate impacts for women and girls specially, there is a perceived implicit impact (positive or negative). For example, given that women are the majority of health care workers (HCWs), responses that target HCWs stand to benefit women by mitigating the impacts for women in this role.

Explicit: Here women and/or girls are called out specifically as a target for the response.

(-) denotes no explicit or implicit consideration of impacts that might affect women and girls in the COVID-19 response plans reviewed.
